# Formation of nanoscale vapour films governing thermal resistance in amorphous ice

**DOI:** 10.1038/s42004-026-02177-2

**Published:** 2026-09-01

**Authors:** Yizhi Liu, Tobias Eklund, Aigerim Karina, Svenja Hövelmann, Robert P. C. Bauer, Seonju You, Claudia Goy, Nele N. Striker, Alexander Gierke, Kenneth Hess, Kai Schlage, Lars Bocklage, Fernando Ardana Lamas, Hao Wang, Yifeng Jiang, Yohei Uemara, Chris Milne, Kyung Hwan Kim, Bridget Murphy, Felix Lehmkühler, Peter Zalden, Katrin Amann-Winkel

**Affiliations:** 1 Max Planck Institute for Polymer Research, Mainz, Germany; 2 Institut für Physik, Johannes Gutenberg-Universität Mainz, Mainz, Germany; 3 European XFEL, Schenefeld, Germany; 4 Department of Chemical Physics, Fysikum, Stockholm University, Stockholm, Sweden; 5 Institute of Experimental and Applied Physics, Kiel University, Kiel, Germany; 6 Ruprecht Haensel Laboratory, Deutsches Elektronen-Synchrotron DESY, Hamburg, Germany; 7 Deutsches Elektronen-Synchrotron DESY, Hamburg, Germany; 8 Department of Chemistry, POSTECH, Pohang, Republic of Korea

**Keywords:** Surfaces, interfaces and thin films, Phase transitions and critical phenomena

## Abstract

Among the many anomalous properties of water, the formation of amorphous ice is one of its most intriguing phenomena. When water vapour is deposited at very low temperatures, it forms an amorphous solid known as amorphous solid water (ASW). Here, we combine X-ray free-electron laser pump–probe diffraction, continuum heat-transfer modelling, and molecular dynamics simulations to investigate the transient thermal response of ASW-films on a platinum substrate. Picosecond laser pulses heat the Pt from 100 K to ~700 K and maintain elevated temperatures for several nanoseconds. Contrary to expectations based on diffusive thermal transport, the hundreds-nanometer-thick ASW layer shows no detectable structural change within tens of nanoseconds. Simulations suggest a mechanism in which a nanometric vapour layer spontaneously forms at the interface, suppressing thermal contact and insulating the ice. The experimental data are consistent with a nanometric (~6 nm) interfacial gap, indicating that the anisotropic scattering originates from a vapour-nucleated interfacial layer. This finding highlights a general non-equilibrium mechanism of interfacial thermal decoupling that may extend beyond the specific ASW–metal system studied here.

**Figure:**
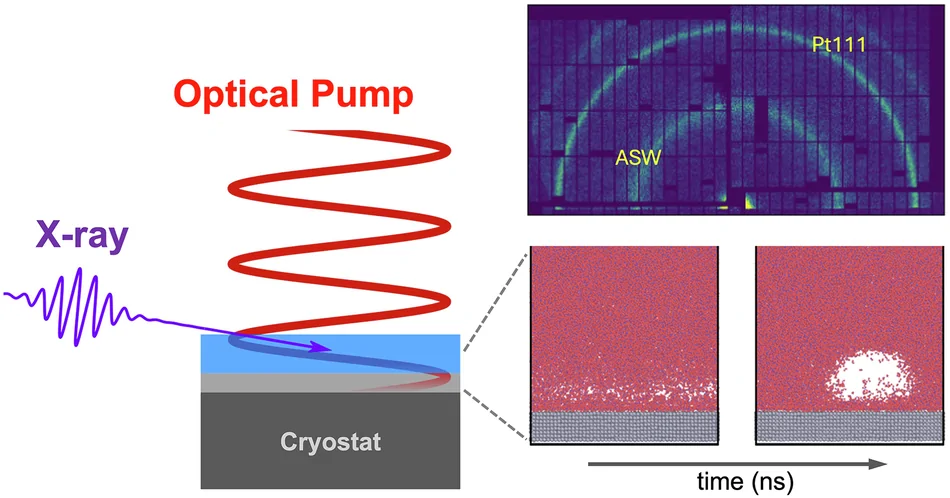

## Abstract (web summary)

Theoretical work has examined non-equilibrium interfacial transport between amorphous solid water (ASW) and metal substrates at the nanosecond timescale, but experimental insights into whether ultrafast interfacial breakdown or decoupling can suppress energy flow across a metal–ASW interface remain scarce. Here, the authors use X-ray free-electron laser pump–probe diffraction, continuum heat-transfer modelling and molecular dynamics simulations to study the transient thermal response of ASW films on a metal substrate, providing insights into non-equilibrium interfacial transport on nanosecond timescales and reporting ultrafast interfacial decoupling via a vapour-nucleated interfacial layer that suppresses energy transfer at the ASW–metal interface.

## Introduction

Nanoscale interfacial heat flow plays a central role in technologies ranging from laser–material processing and magnetic data storage to nanoscale electronics and heterogeneous catalysis, where energy is injected into solids on sub-nanosecond time scales. In these settings, the thermal response is often governed not by bulk properties but by the structure and evolution of the interface itself^1–4^. At ultrafast excitation rates, energy can be deposited faster than it can be redistributed within the adjacent material, producing a transient temperature imbalance across the interface. Under these conditions, the interfacial region enters a transient non-equilibrium state, such that the steady-state values of interfacial thermal conductance are no longer adequate, even though the underlying framework for heat transport remains valid. This behaviour is evident in recent ultrafast pump–probe studies across different material systems: in FeRh/MgO, a photoinduced structural transition reduces the interfacial thermal conductance by nearly a factor of five within ~90 ps^5^, whereas at Au/hBN interfaces, hyperbolic phonon–polaritons facilitate an interfacial heat-transfer channel that sharply increases the effective thermal coupling compared with conventional phonon-mediated conduction^6^. These examples underscore that, under ultrafast excitation, interfacial heat transfer can deviate sharply from steady-state phonon-mediated expectations, responding instead to transient structural evolution and non-equilibrium energy carriers.

Water provides a sensitive platform for exploring ultrafast structural response because its local structure and phase behaviour respond strongly to rapid temperature perturbations^7–17^. Upon deep supercooling, water exhibits pronounced anomalies in compressibility^18,19^ and heat capacity^20–22^ that point to a possible liquid–liquid phase transition (LLPT) between high-density and low-density liquid states^23^. However, below 235 K, crystallization proceeds on microsecond timescales^24–27^, rendering the ~150–235 K region—commonly referred to as the “no-man’s-land” at ambient pressure—effectively inaccessible for equilibrium studies of the metastable liquid. Ultrafast excitation has opened new pathways for transiently accessing this regime^25,26,28–34^. Rapid infrared heating combined with femtosecond X-ray probing has shown that low-density liquid domains can emerge within tens of nanoseconds before crystallizing on the microsecond scale, providing direct time-resolved evidence for liquid–liquid separation in amorphous ice^28^. Structural signatures of an LLPT have been revealed through two complementary non-equilibrium routes: rapid heating of high-density amorphous ice (HDA) under pressure, which produces a high-density liquid that subsequently converts into a low-density liquid^26^, and ultrafast isochoric heating of low-density amorphous ice (LDA), which yields transient low-density liquid domains on nanosecond timescales before crystallization^28^. Another form of LDA, also called amorphous solid water, can be formed by vapour deposition on cold (<130 K) substrates^35,36^, a process naturally occurring on, e.g., interstellar dust grains^37,38^. As such it can be prepared in nm-thick layers. We here extend the X-ray pump–probe experiments to ASW films by an indirect heating approach in which a buried metal layer acts as an ultrafast optical transducer that rapidly heats the overlying ASW, using picosecond laser pulses. Similar metal-mediated heating schemes have been used previously to study desorption and crystallization kinetics of ASW films^28^, using ns-laser pulses and spectroscopic probes^39^. X-rays, however, are directly sensitive to structural changes in the amorphous network and provide insights into the nanoscale pathways by which amorphous solid water responds when heated through a buried metal–ice interface.

Open research questions are how an ultrafast heat pulse delivered from a metal substrate reshapes the interfacial region, alters mechanical coupling, or drives transient structural reorganization in the ASW film. Conventional descriptions of interfacial heat transfer treat the thermal boundary conductance as a fixed material property determined by phonon or electron transmission across a static atomic interface^40,41^. Such models hold under steady-state or quasi-equilibrium conditions. However, at nanometer and nanosecond scales, these assumptions fail: when energy is delivered faster than the interface can relax, the boundary becomes dynamically reconfigured, and the notion of a time-independent conductance no longer applies^42–44^. Although recent theoretical work has begun to examine this regime of non-equilibrium interfacial transport^42–45^, there is still no direct experimental insight into whether ultrafast interfacial breakdown or decoupling can suppress energy flow across a metal–ASW interface^5^. Clarifying whether such ultrafast interfacial failure limits energy flow is essential, because these effects are expected to dominate heat transport under pump–probe conditions. To address this question, we combine time-resolved X-ray free-electron laser (XFEL) diffraction^46^, continuum heat-transfer modelling, and molecular dynamics (MD) simulations to directly follow the non-equilibrium evolution of a metal–ice interface under nanosecond thermal excitation.

We show that interfacial heat transfer under ultrafast heating conditions can be strongly altered by transient changes in the interface itself. Using amorphous ice vapour-deposited on a platinum surface at 100 K as a model system, we observe that rapid heating of the metal to several hundred degrees Celsius can leave the overlying ice layer largely unaffected on nanosecond timescales. Our results indicate that this unexpected behaviour arises from the rapid formation of a nanometric low-density vapour layer at the metal–water interface, which transiently suppresses heat transfer across the boundary. Such interfacial decoupling is not captured by simple diffusive heat-transfer descriptions. By combining time-resolved X-ray scattering experiments with atomistic simulations, we show that ultrafast heating can trigger interfacial density depletion and the emergence of an interfacial low-density layer, providing a microscopic mechanism for the observed thermal insulation.

## Results

### XFEL experimental observations

**Fig. 1:**
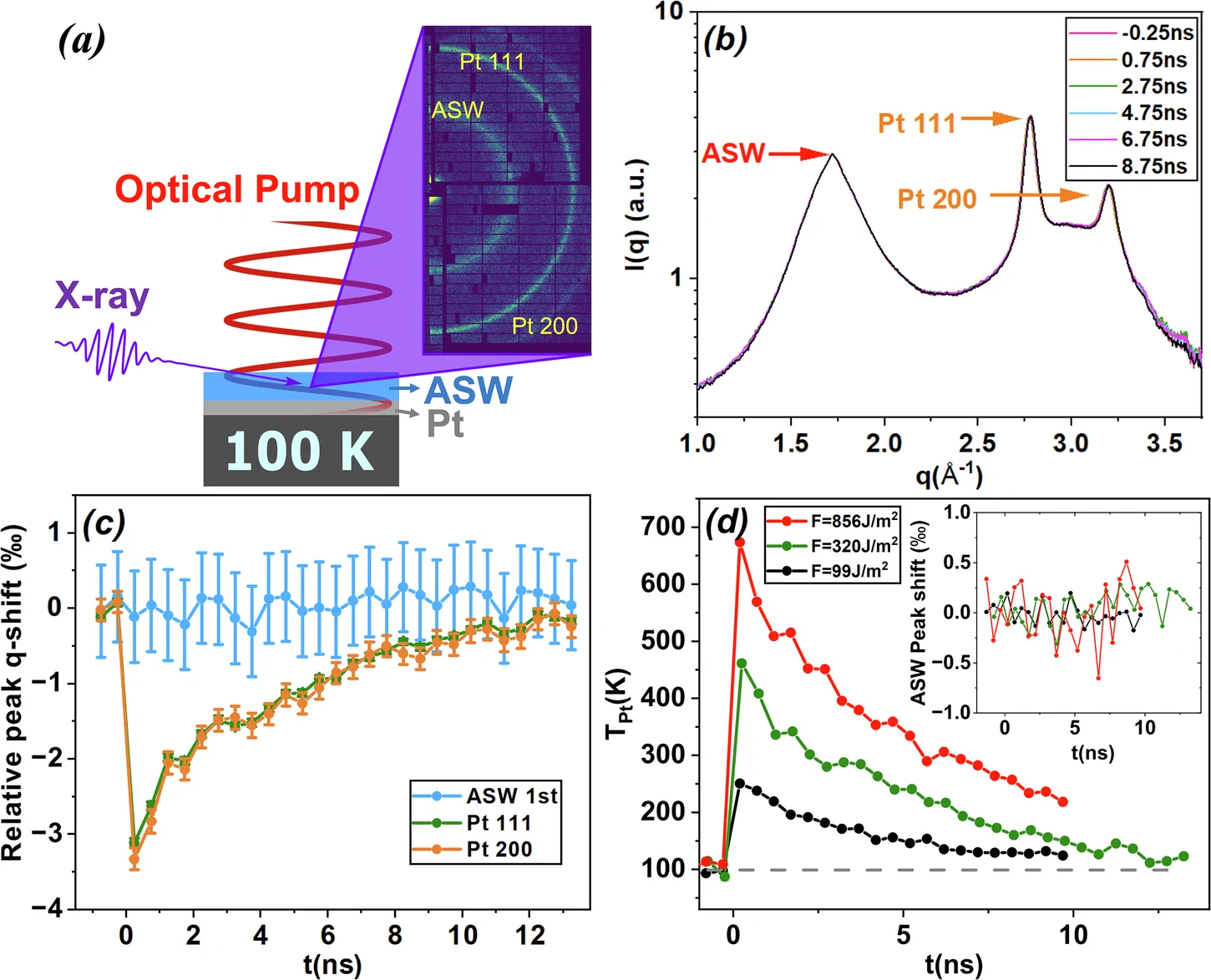
Diffraction response of the Pt/ASW multilayer following IR-laser excitation. **a** Schematic of the pump–probe setup used at the European XFEL, including detector image. An infrared pulse heats the 30 nm Pt film, and time-resolved X-ray scattering in reflection geometry monitors the structural evolution of the 100 nm ASW layer. **b** Integrated X-ray scattering intensity I(q) of the ASW/Pt multilayer at selected pump–probe delays. Each scan step is averaged over 5 s of measurement time, corresponding to 25 X-ray pulses (50 fs duration each) at a repetition rate of 5 Hz. **c** Relative peak-position shifts of the Pt (111) and (200) reflections and the ASW main peak as a function of pump–probe delay at a pump fluence of 320 J/m². The Pt peaks shift to lower *q* due to transient thermal expansion, whereas the ASW peak remains unchanged within experimental uncertainty. The temporal spacing between consecutive data points is Δ*t* = 0.5 ns. Each data point was averaged over *n* = 147, 148, 149, or 150 individual X-ray pulse trains (the exact number contributing to each delay point is provided in the Source Data), depending on the number of successfully recorded pump–probe measurements at the corresponding delay. Error bars represent standard errors of the relative peak shifts, calculated by propagating the peak position uncertainties extracted from the fit covariance matrix. **d** Time-dependent Pt temperature extracted from the diffraction peak shift using the Pt thermal expansion. The inset shows the relative peak shift of the first ASW diffraction peak, which remains below 0.05% within 10 ns.

Amorphous solid water (ASW) films were prepared by vapour deposition of ultrapure H₂O onto a platinum substrate maintained at 100 K under high vacuum (1.3 × 10^−^^5^ mbar). The substrate consisted of a 30 nm Pt layer on an oxidized Si wafer (SiO_2_ surface layer, see details under Methods). The resulting ASW films had a thickness of approximately 100 nm (see Methods). Pump–probe experiments were performed at the FXE instrument at European XFEL using an infrared pump/X-ray probe setup (Fig.1a). The infrared laser pulses, of wavelength 800 nm and 0.6 ps (chirped) pulse duration, were primarily absorbed by the Pt substrate, which was transiently heated and subsequently transferred heat to the ASW film through thermal conduction.

Time-resolved X-ray scattering at 9.3 keV (50 fs pulses) in a grazing-incidence wide-angle X-ray scattering (WAXS) geometry (using the LPD detector^47^) simultaneously tracks both the Pt lattice and the ASW structure (Fig. 1b) by following the transient shifts of the Pt Bragg peaks and the evolution of the first diffraction peak of ASW. The ASW scattering maximum is located at *q *≈ 1.7 Å^−1^, while the Pt (111) and (200) reflections appear at *q* ≈ 2.8 Å^−^^1^ and $3.3~{{{{\AA }}}}^{-1}$, respectively (Fig. 1b). The following picture emerges. Upon infrared laser excitation, the Pt diffraction peaks exhibit a clear shift toward lower q, consistent with thermal expansion of the lattice, whereas the main peak of amorphous solid water (ASW) remains nearly unchanged (Fig. 1c). To isolate the laser-induced structural response, the unexcited diffraction pattern was subtracted from the laser-excited one to obtain the differential diffraction intensity (${\Delta I=I}_{{{{\rm{on}}}}}-{I}_{{{{\rm{o}}}}{{{\rm{ff}}}}}$, Figure S1). This revealed pronounced peak shifts at the Pt reflections but negligible changes in the ASW signal (Fig. 1c), confirming that the heating primarily affects the metallic substrate—a response incompatible with any Fourier-based model using thermal parameters or static interfacial resistances.

To quantify the transient lattice expansion, the peak positions were extracted from Voigt profile fits. From the shifts of the Pt (111) and (200) peaks, we computed the corresponding temperature increase (see Methods) (Fig. 1d). The time-dependent temperature evolution of the Pt layer is measured by repeatedly pumping and probing the same spot while scanning the pump–probe delay. The X-ray probe operates at 10 Hz, while the IR pump is applied at 5 Hz (i.e., every second probe pulse). In a series of measurements, the pump fluence was adjusted to reach peak Pt temperatures of approximately 250 K, 450 K, and 700 K at ~0.25 ns after excitation. The Pt surface then gradually cools to its initial state within 10 ns. Throughout this period, however, the position of the ASW peak remains constant within experimental uncertainty (<0.05%), indicating that the ASW structure is unaffected by the transient heating of the substrate. Additionally, in a separate fluence-scan measurement where the Pt substrate reached temperatures approaching ~1200 K (Figure S2), the ASW peak position and line shape still showed no detectable change within our experimental resolution.

The invariance of the ASW peak position and line shape over such a wide temperature range demonstrates that only a negligible amount of heat is transferred into the ASW within the 10 ns timescale. This pronounced thermal decoupling indicates an unexpectedly large interfacial thermal resistance between the Pt substrate and the amorphous ice. These results highlight the complex and still poorly understood thermal behaviour of nanoscale amorphous ice, where both spatial and temporal non-equilibrium effects can give rise to such anomalous interfacial phenomena.

### Heat conduction modelling

**Fig. 2:**
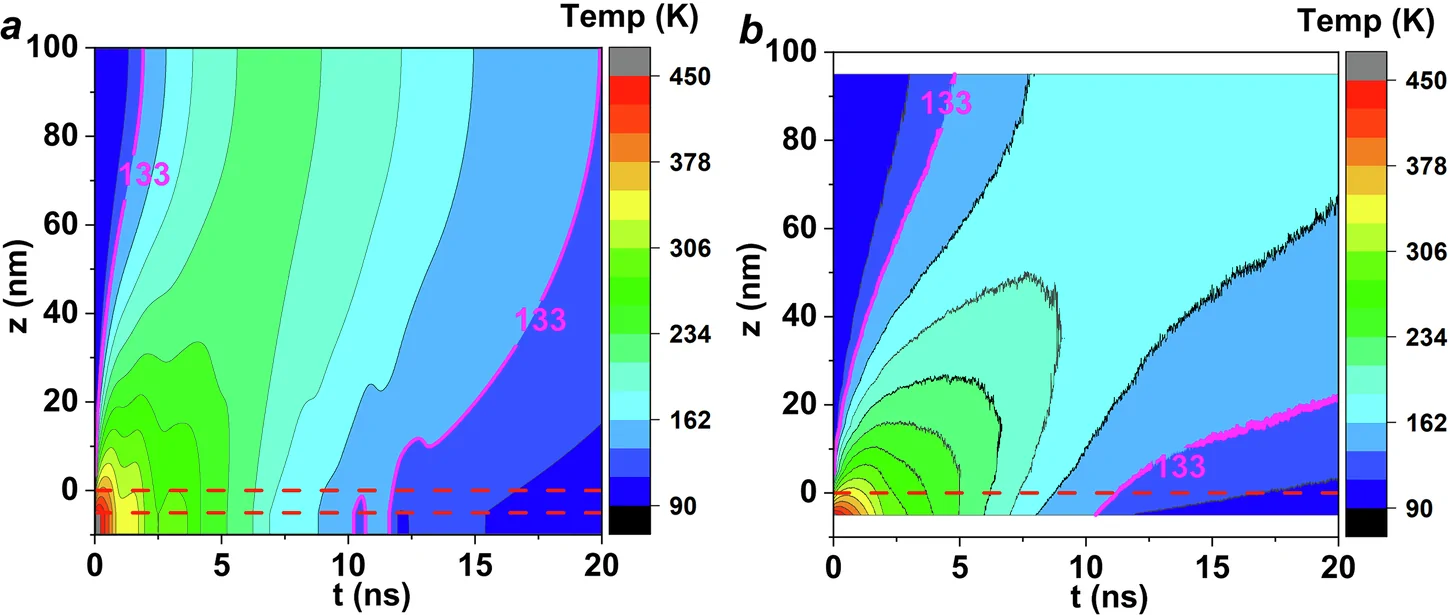
Temperature profiles (*z*, *t*) of the ASW film under different Pt heating protocols. Continuum and molecular-dynamics simulations illustrating heat transport across the 100-nm ASW layer. **a** Continuum heat-conduction simulation based on the one-dimensional thermal diffusion equation for a Pt substrate heated to 450 K and subsequently cooled. The temperature front propagates from the Pt–ASW interface into the film. The thick magenta contour denotes a reference isotherm at *T* = 133 K, close to the experimentally reported glass-transition temperature of ASW. Here, the isotherm is shown only as a reference to indicate the propagation of thermal fronts, rather than a precise glass transition under non-equilibrium conditions. The red dashed lines delimit a 5-nm interfacial region at the Pt–ASW boundary, within which an effective interfacial thermal conductance is applied in the continuum model. **b** Molecular dynamics simulations using TIP4P/2005 show a similar spatiotemporal evolution of temperature across the ASW film. The same 133 K isotherm is plotted for direct comparison with the continuum model. The red dashed line marks the position of the Pt–ASW boundary as defined in the atomistic model.

To evaluate whether heat conduction alone could account for the observed minimal structural response of ASW, we modelled the transient temperature evolution across a Pt–ASW bilayer using both continuum heat diffusion and atomistic molecular dynamics (MD) simulations (Fig. 2). The continuum and MD models used simplified Pt–ASW stacks with thin Pt layers beneath a ~100-nm ASW film. In this framework, the *z*-coordinate is defined such that *z* = 0 corresponds to the Pt–ASW interface, with *z* > 0 extending into the ASW layer. Because the Pt temperature was taken directly from the experimentally measured transient temperature of the metal film, the boundary condition effectively represents the interfacial temperature experienced by the ASW layer and already incorporates the net effect of heat dissipation into the underlying Si substrate. In MD simulations, the ASW was first prepared by rapid quenching from 350 K to 100 K in a large-scale simulation box (6 × 6 × 150 nm³). The subsequent heating was driven by coupling the Pt atoms to a thermostat that followed the experimental ${{T}}_{{{Pt}}}(t)$, while the ASW evolved freely without external temperature control (Methods). We used the TIP4P/2005 water model^48^ for the main investigation, while additional simulations using the SPC/E model^49^ are provided in the Supplementary Information (Figure S3–4). Both models yield qualitatively consistent results for the interfacial behaviour discussed here.

Despite their different levels of description, both continuum heat diffusion and MD simulations yield nearly identical thermal-penetration dynamics. Using experimentally measured thermophysical properties for both materials—namely, a thermal conductivity of *k*_*Pt*_ = 72 W m^-1^ K^-1^^50^ for the Pt layer and ${k}_{{ASW}}=0.6 \, {{{\rm{W}}}}{{{{\rm{m}}}}}^{-1}{{{{\rm{K}}}}}^{-1}$ for amorphous ice. The latter value is obtained from transient hot-wire measurements^51,52^ and is comparable in magnitude to that of liquid water, with only weak temperature dependence under low-pressure conditions. Heat transfer across the Pt–ASW interface is characterized by a Kapitza conductance *G*: J=GΔT. Based on literature values from both experiments and molecular-dynamics simulations, we adopted a representative interfacial thermal conductance of *G* ≈ 130 MW m^−2^ K^−1^ for the Pt–ASW interface^53^. Given the partial wettability of water on Pt (Figure S5), this conductance provides a physically reasonable estimate for our system. In the numerical implementation, the interfacial resistance was introduced by assigning a 5-nm interfacial layer an effective conductivity *k*_*int*_* = G·L*_*int*_ *≈ *0.65 W m^−1^ K^−1^, ensuring that the correct Kapitza resistance is imposed at the Pt–ASW boundary. All thermophysical parameters, including the interfacial thermal conductance, are taken from literature values as detailed in the Methods section^50–57^.

With these parameters, the continuum model predicts that the 133-K isotherm—close to the commonly reported glass-transition temperature (*T*_g_) of amorphous ice^31,58^ —sweeps through the entire 100-nm ASW film within ~2 ns (Fig.2a). Consistently, the MD simulations show similar rapid propagation of energy from the Pt substrate across the ASW film on nanosecond timescales (Fig. 2b). Here, we note that 133 K is not the glass transition temperature in the MD simulations, as *T*_g_ is strongly dependent on the model and thermal history and is not well defined under non-equilibrium conditions. Instead, the isotherm is used as a reference to compare the propagation of thermal fronts with the continuum heat conduction model. Together, these results indicate that, under our experimental heating conditions, conventional heat conduction can rapidly raise the temperature of the entire ASW layer into a regime where structural relaxation would be expected within a few nanoseconds.

**Fig. 3:**
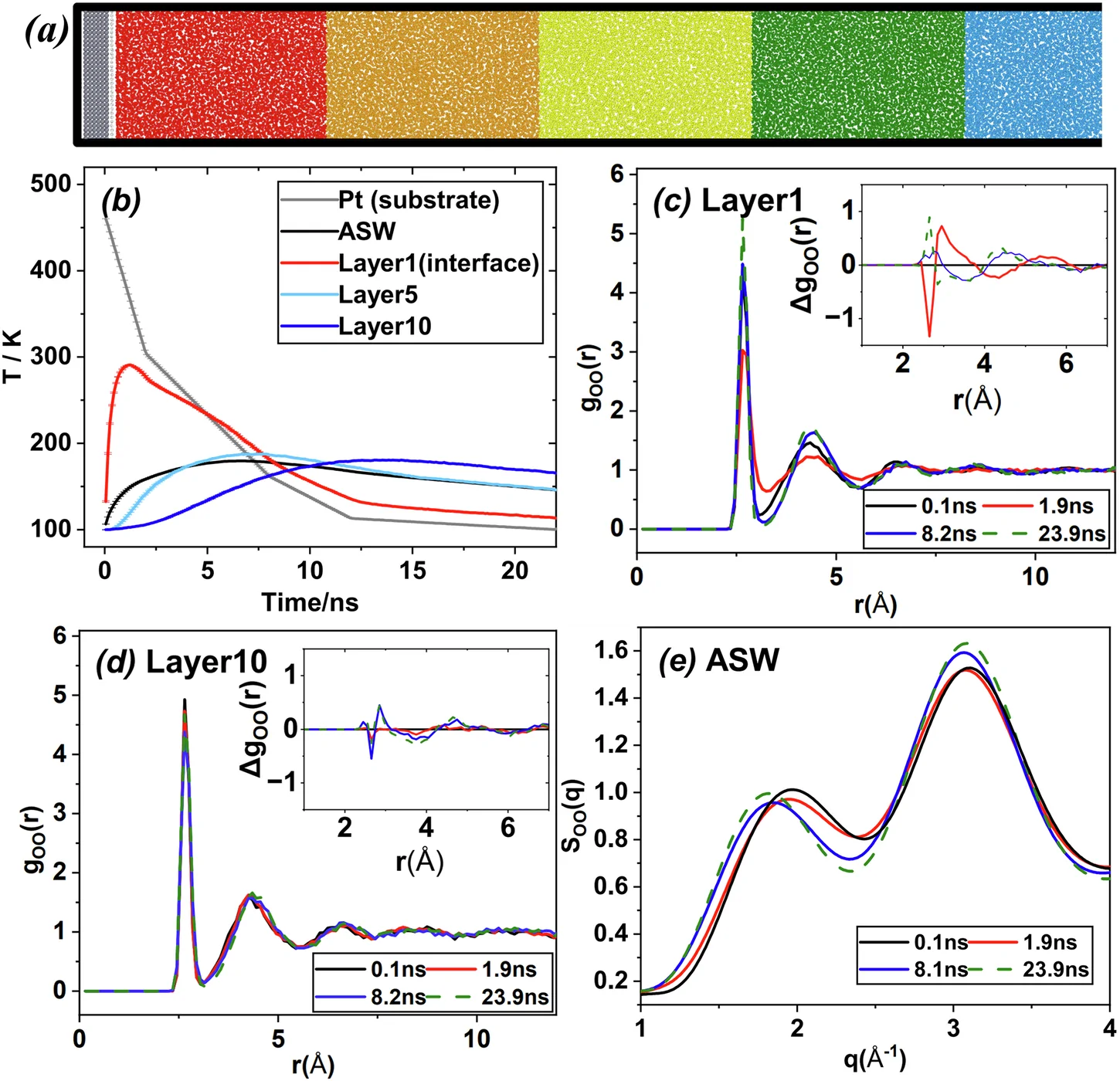
Layer-resolved structural evolution of ASW during MD heating simulations. **a** Illustration of the simulation box and division into layers, with the first layer (near the Pt interface) highlighted in red. **b** Time evolution of temperature *T*(*t*) for the Pt substrate (grey), the ASW film (black), and representative layers (Layer 1, Layer 5, and Layer 10). The lines show the mean of (*n* = 4) independent molecular dynamics simulations, and the shaded bands indicate the standard error of the mean (s.e.m.). The rapid temperature rise of the interfacial layer indicates local melting near Pt, while layers farther from the interface remain low temperature for several nanoseconds. Although this suggests that only the near-interface region melts, preparing ASW films much thinner than ~100 nm is experimentally impractical: below ~50–100 nm, the X-ray scattering signal becomes too weak, and background deposition dominates the diffraction pattern. Four representative delay times are shown to illustrate the structure evolution from the interface to the bulk. Layer-resolved $$ {g}_{{OO}}(r)$$ for the first (near-interface) (**c**) and tenth (interior) layers (**d**), showing the structural evolution at four representative delay times. Insets display the differential curves $${\Delta g}_{{OO}}(r)$$ relative to the initial state to highlight subtle changes in the first and second coordination shells. **e** Oxygen–oxygen structure factor *S*_*OO*_(*q*), averaged over all layers, shown at the same representative delay times as in (**c**, **d**).

For spatially resolved analysis, the ASW film in the MD simulations was divided along the surface normal (*z* direction) into ten layers of equal thickness (10 nm each), with layer 1 in direct contact with the Pt substrate (Fig. 3a). At early times (*t* < 1 ns), the temperature distribution is strongly heterogeneous (Fig. 3b). The interfacial layers rapidly heat above 133 K and exhibit clear liquid-like structural signatures—broadening of the first peak and suppression of the second-shell maximum in ${g}_{{OO}}(r)$ (Fig. 3c). In contrast, the topmost layer (layer 10), farthest from the interface, retains the narrow first-peak and well-defined second-shell structure characteristic of glassy ASW (Fig. 3d), showing essentially no structural evolution at this early stage.

As the system evolves (*t* = 8–12 ns), the temperature gradient across the ASW film gradually decreases but does not vanish; even at later times (*t* > 12 ns), a residual drop of ~100 K persists across the 100-nm slab, indicating that the system remains far from thermal equilibrium with the substrate throughout the simulation. Consequently, the oxygen–oxygen structure factor ${{S}_{OO}}({q})$ of the film (Fig. 3e) reflects a non-equilibrium mixture of liquid-like and glassy regions rather than a single homogeneous state. The position of the first diffraction peak shifts from 1.935 to 1.824 Å^−1^—a displacement of 0.111 Å^−1^ (5.7%), far exceeding the ≈0.05% experimental precision within which no statistically significant change is observed—demonstrating that heating in the simulation induces pronounced structural evolution that is absent in the measurements.

The discrepancies between experiment and simulation likely arise from suppressed heat transfer across the Pt–ASW interface under the applied heating conditions. The effective interfacial thermal conductance, *G*, may therefore be significantly lower than reported for metal–water interfaces. This observation points to the presence of an additional interfacial mechanism beyond conventional diffusive heat transport.

### Nano-sized vapour-film formation

**Fig. 4:**
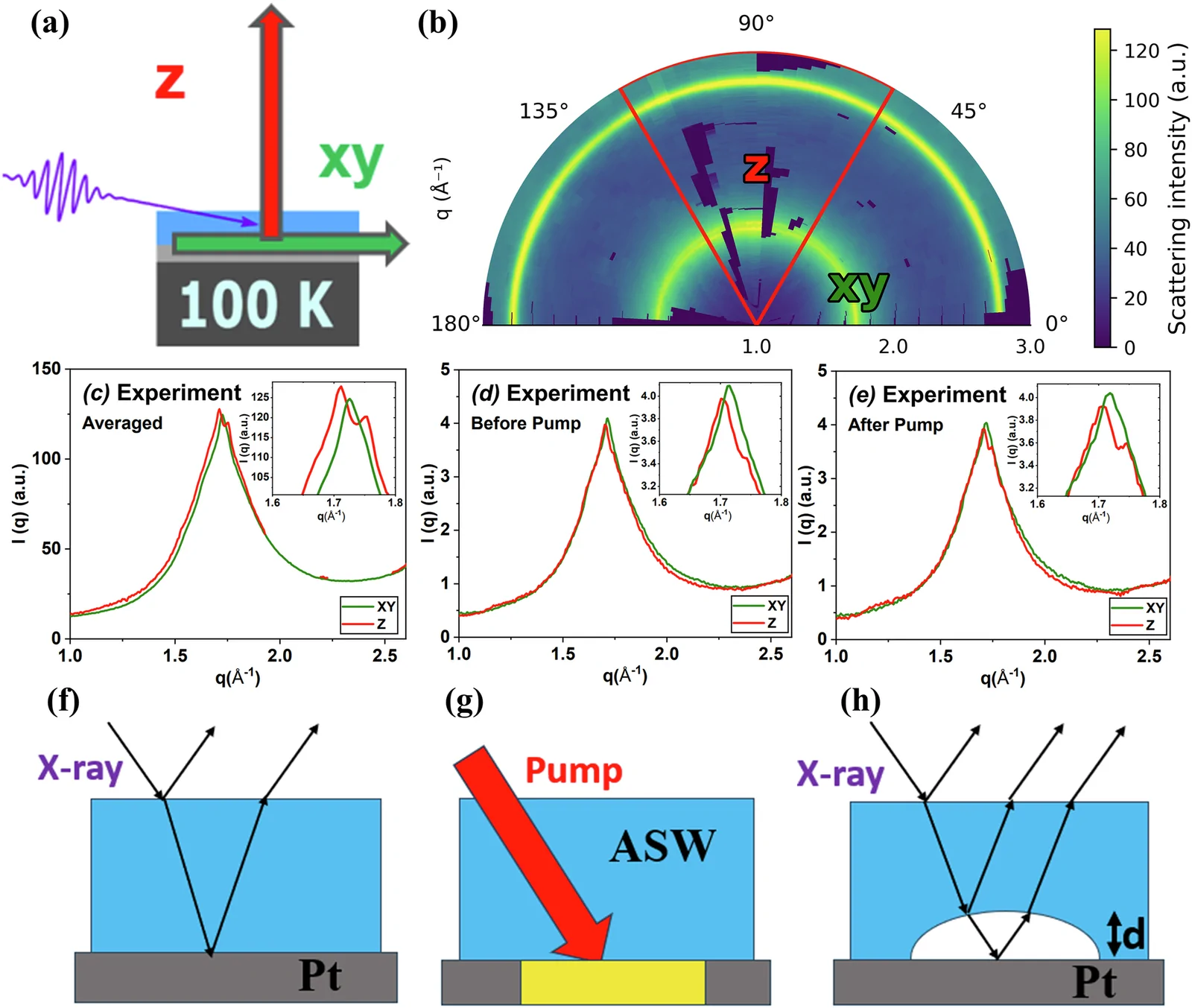
Structural anisotropy and interference signature of the interfacial vapour layer. **a** Schematic of the X-ray scattering geometry in real space and the corresponding 2D GIWAXS pattern in reciprocal space (**b**), averaged over the same pump–probe conditions. The integration regions used to compute *I*(*q*_z_) and *I*(*q*_xy_) are indicated: *I*(*q*_z_) is obtained from the red-highlighted sector along the specular direction, while *I*(*q*_xy_) is obtained by integrating over the remaining angular regions in the detector plane. **b**, **c** Experimentally measured *I*(*q*_z_) and *I*(*q*_xy_) at a pump fluence of 99 J/m², obtained by averaging all X-ray pulses acquired during the delay scan, including both pump-on and pump-off shots. Insets show magnified views of the peak apex to highlight subtle variations. **d**, **e** Representative data from the multi-pulse mode. Each sequence contains one pump pulse (320 J/m²) followed by 30 X-ray probe pulses. **d** Shows a sequence recorded before the pump, and (**e**) shows a sequence recorded after the pump, with the two types of sequences alternating during acquisition. Insets highlight the peak regions for clarity. **f**–**h** Schematic illustration of the scattering mechanism. Without a vapour gap, X-rays reflect mainly at the Pt–ASW interface. With a nanometer-scale vapour gap, X-rays reflect from both the Pt–gap and gap–ASW interfaces, creating an optical path difference d that leads to an interference period Δ*q*_z_ = 2π/*d*.

The grazing-incidence X-ray scattering geometry (GIWAXS) presented here provides a unique opportunity for two-dimensional characterization of the ASW film, both perpendicular and parallel to the underlying surface. Based on the scattering geometry (Fig. 4a), the diffraction signal can be decomposed into in-plane (${q}_{{xy}}$) and out-of-plane (${q}_{z}$) components relative to the interface (Fig. 4b). We observe that the X-ray scattering patterns along the surface normal (Fig. 4c) exhibit a pronounced anisotropy. At an incident pump fluence of 99 J/m^2^ across the projected area on the sample, the WAXS intensity averaged over all shots shows a clear double peak only in the out-of-plane (${q}_{z}$) direction (Fig. 4c), whereas the in-plane (${q}_{{xy}}$) signal remains single-peaked. A similar anisotropic response is observed at higher pump fluences (Figure S6).

To assess the pump-induced structural response in more detail, we performed measurements at an incident (projected) pump fluence of 320 J m^−^² using sequences of 30 X-ray probe pulses, separated by ~440 ns. This single-pump multiple-probe scheme makes use of the unique possibilities of the European XFEL (see SI). Comparing the signals before the pump (Fig. 4d) and after the pump (Fig. 4e, 5 ns–400 µs), we find that the ASW peak remains single-peaked in the ${q}_{{xy}}$ direction throughout. In contrast, a double peak appears along ${q}_{z}$ immediately after pumping and persists over the entire measurement window. The splitting, with a characteristic separation of Δ*q* ≈ 0.05 Å^−1^, is reproducible across multiple pump–heating sequences. For reference, in the absence of a pump—when the sample is exposed only to the X-ray probe—no double peak is observed in either direction (Figure S7). The observed peak splitting can be understood as the reciprocal-space signature of a nanoscale separation developing near the Pt–ASW interface (Fig. 4f–h). In this scattering geometry, two nearby interfaces act as partially reflecting boundaries and generate an intensity modulation whose spacing scales inversely with their separation. Using the approximate relation Δ*q* ≈ π/*d*, which captures the spacing between the first pair of extrema for a two-interface system, a uniform 100 nm ASW film would produce a modulation spacing of only Δ*q*_z_ ≈ π/*d* ≈ 0.003 Å^−1^—far below the instrumental resolution and therefore unobservable. If a nanometric interfacial vapour layer forms, however, the relevant separation becomes much smaller, leading to a substantially larger modulation spacing. The experimentally observed splitting of Δ*q*_z_ ≈ 0.05 Å^−1^ corresponds to a characteristic separation of *d* ≈ 6 nm, suggesting the transient formation of a nanoscale interfacial vapour layer at the Pt–ASW boundary.

**Fig. 5:**
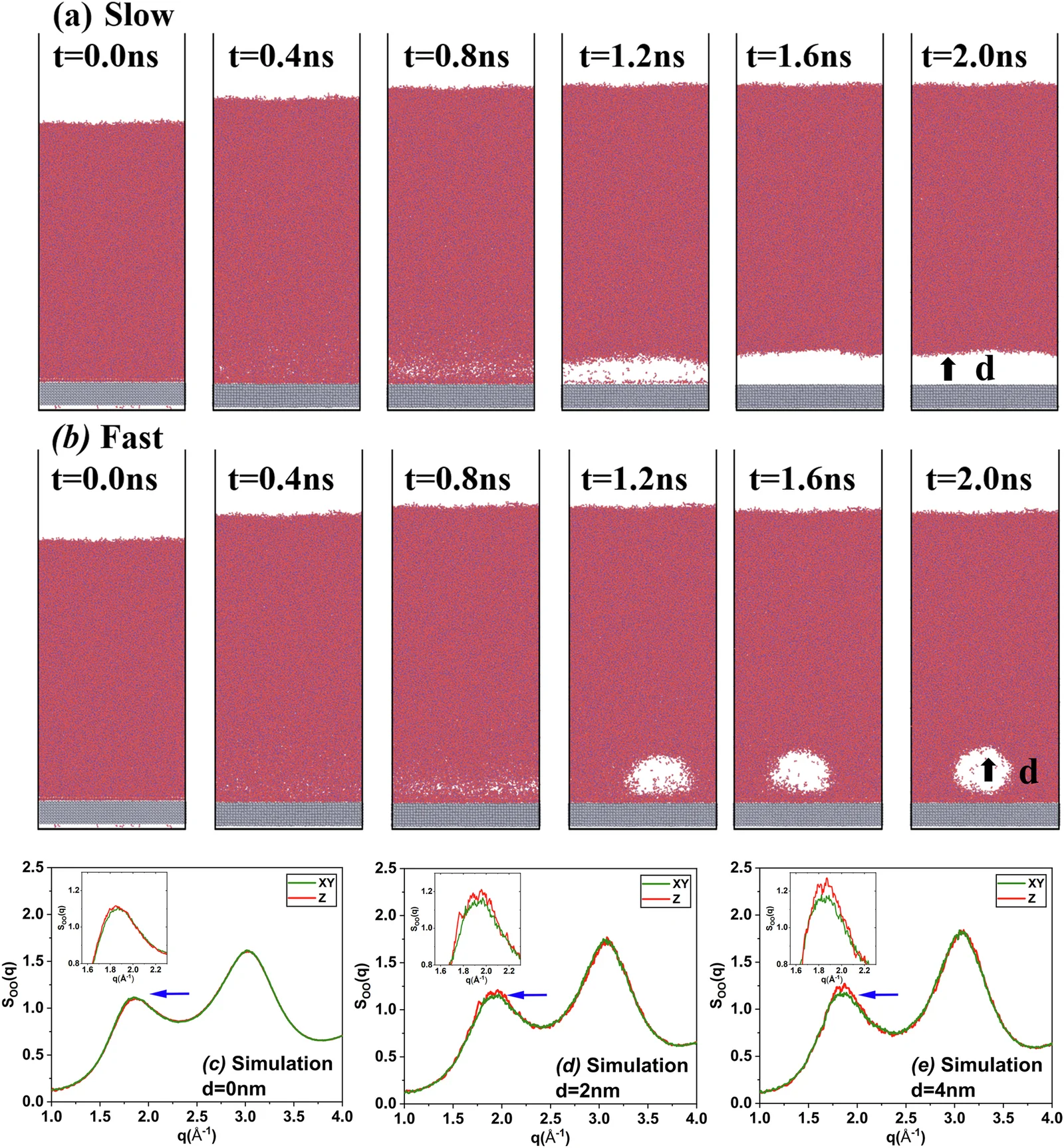
Molecular dynamics evidence and experimental confirmation of vapour-layer formation at the Pt–ASW interface. **a**, **b** MD snapshots at *t* = 0, 0.4, 0.8, 1.2, 1.6, 2.0 ns showing the structural evolution of the Pt–ASW boundary following an impulsive heating of the Pt slab to 1000 K. Two cooling protocols are compared: **a** slow quench (10 ns) and (**b**) fast quench (1 ns). In both cases, a nanometric low-density vapour layer forms at the interface within <1 ns and remains stable throughout the relaxation period, consistent with the experimentally observed thermal decoupling between the hot Pt substrate and the structurally cold ASW film. The arrow d indicates the thickness of the low-density layer. Red and blue spheres denote oxygen and hydrogen atoms. MD-calculated scattering profiles $S({q}_{z})$ and $S({q}_{{xy}})$ for three interfacial conditions: **c** no gap, (**d**) a 2 nm vapour gap, and (**e**) a 4 nm vapour gap. The insets enlarge the first diffraction peak to highlight the anisotropy between the out-of-plane, and in-plane scattering profiles, which becomes more pronounced as the vapour gap increases. A larger gap produces stronger splitting in $S({q}_{z})$ due to the increased phase delay between reflections at the two interfaces. The 2 nm gap corresponds to a peak splitting of Δ*q* ≈ 0.08 Å^−1^, while the 4 nm gap yields Δ*q* ≈ 0.06 Å^−1^, consistent with the inverse relation between gap thickness and interference spacing.

To explore the microscopic origin of the interfacial separation suggested by the GIWAXS measurements, we performed a second set of atomistic simulations specifically designed to resolve structural evolution at the Pt–ASW boundary. In this interfacial-geometry MD model, a larger in-plane area and a reduced film thickness were used to allow interfacial rearrangements to develop. The Pt slab was driven with a nanosecond relaxation profile similar to that in the experiment, with peak temperatures set to 600K-1000 K, chosen as a representative high-temperature condition within the experimentally observed range of ${T}_{{Pt}}(t)$ (200–1200 K; see SI).

The simulation box (10 × 10 × 33 nm^3^) comprised a 2 nm Pt slab and an 18 nm ASW film. Here, we again use the TIP4P/2005 water model. Additional simulations using the SPC/E model are provided in the SI (Figures S8-9); both models yield qualitatively consistent interfacial behaviour. In the experiment, the Pt surface is impulsively heated to temperatures between 200 and 1200 K and subsequently relaxes back to ~100 K within ~10 ns, while the ASW layer shows no measurable structural response. To mimic this thermal evolution, the Pt slab in the simulation was heated to 1000 K at *t* = 0 ns and then cooled to 100 K over 10 ns, approximating the experimental relaxation dynamics. Because the simulated ASW film is thinner than the experimental ~100 nm layer, a thermostat was applied only to the top ~2 nm of the simulated film to represent a far-field boundary that maintains the bulk-like region near 100 K. The remaining ~16 nm of the ASW—including the interfacial region—evolved without external heat exchange. Under these conditions, significant heat transfer from the Pt would lead to a temperature rise propagating upward into the film.

When the Pt surface reaches 1000 K, a nanometric interfacial low-density layer forms at the Pt–ASW boundary within ~0.4 ns (Fig. 5). In the slow-quench case (Fig. 5a), this nanometric layer persists throughout the relaxation. Because the upper ASW layer remains deeply glassy, molecular mobility is extremely slow. Vapour-phase water molecules generated near the interface collide with the cold ASW and gradually re-adsorb, progressively reducing the vapour density. As the Pt temperature relaxes, this process leads to the emergence of a nanometric interfacial layer separating the Pt and ASW layers, which might also be described as a vacuum-like gap. The limited molecular mobility in the glassy ASW layer prevents rapid structural relaxation of this void, allowing the interfacial gap to persist during the thermal cycle. As a result, the eventual interfacial morphology is largely determined by the subsequent cooling rate of the Pt–ASW system. A fast-quench protocol (Fig. 5b) produces the same rapid (<1 ns) nucleation of the interfacial low-density layer but results in a qualitatively different final morphology: the ASW near the interface becomes porous because the system is quenched before the vapour can fully re-adsorb.

The formation of a nanometric interfacial layer breaks the structural isotropy of the film and produces distinct out-of-plane and in-plane scattering responses. To quantify this effect, we computed direction-resolved structure factors from the microscopic density definition for MD configurations with nominal gap widths of 0, 2, and 4 nm (Figure S10). When no gap is present, S(${q}_{z}$) and S(${q}_{{xy}}$) show nearly identical broad maxima, consistent with an isotropic amorphous structure (Fig. 5c). Introducing a ~ 2 nm cavity leaves the in-plane response essentially unchanged but causes the first peak in S(${q}_{z}$) to split (Fig. 5d). A larger ~4 nm gap further shortens the modulation period and increases the degree of splitting (Fig. 5e).

To clarify this mechanism, we consider a minimal one-dimensional model in which an ASW slab contains a vacuum layer of width d:

$$\rho (z) = \left\{\begin{array}{l}{\rho}_{0}, \quad z < 0 \\ 0, 0 < z < d \\ {\rho }_{0}, \quad z > d\end{array}\right.$$

The out-of-plane structure factor is proportional to the modulus square of the Fourier transform of this profile, yielding $S(q_z)\propto\frac{2\rho_0^2}{q_z^2}\left[1-\cos(q_zd)\right]$ (Methods). Thus, a cavity of thickness d produces a modulation with a characteristic spacing $\Delta {q_z} \sim \pi / {d}$ between neighbouring extrema. In our simulations, the absence of a corresponding double peak in the directional g(r) (Figure S11) indicates that the observed splitting in ${{S}}({q}_{z})$ is dominated by vacuum layer rather than by changes in the local structure. Consistent with this behaviour, a 2 nm gap (Fig.5d) yields Δ*q*_*z*_ = 0.08 Å^−1^, whereas a thicker 4 nm gap produces a smaller splitting (Fig.5e) of Δ*q*_*z*_ = 0.06 Å^−1^. Quantitatively, the product $d\, {\cdot}\, {\Delta} {q}_{z}\approx 1.6-2.4$, which is of the same order as π, suggesting that the observed trend is reasonably captured by an approximate relation $\Delta {q}_{z}\sim \pi / {d}$. While the exact pre-factor depends on the detailed density gradients within the gap, the inverse scaling itself is robust.

The experimental line shape appears smooth and does not display a full oscillatory series. Several experimental factors can suppress higher-order oscillations, including ensemble averaging over many XFEL pulses, spatial averaging over the illuminated sample area where the gap thickness is likely not uniform, limited reflectivity contrast at the boundaries, and finite *q*-resolution. As a result, only the primary modulation remains observable, appearing as a weak peak splitting. Consistent with this interpretation, oscillatory features can indeed be observed in multiple-pulse data and in small-shot averages (Figure S12), but become progressively averaged out as more shots are included.

**Fig. 6:**
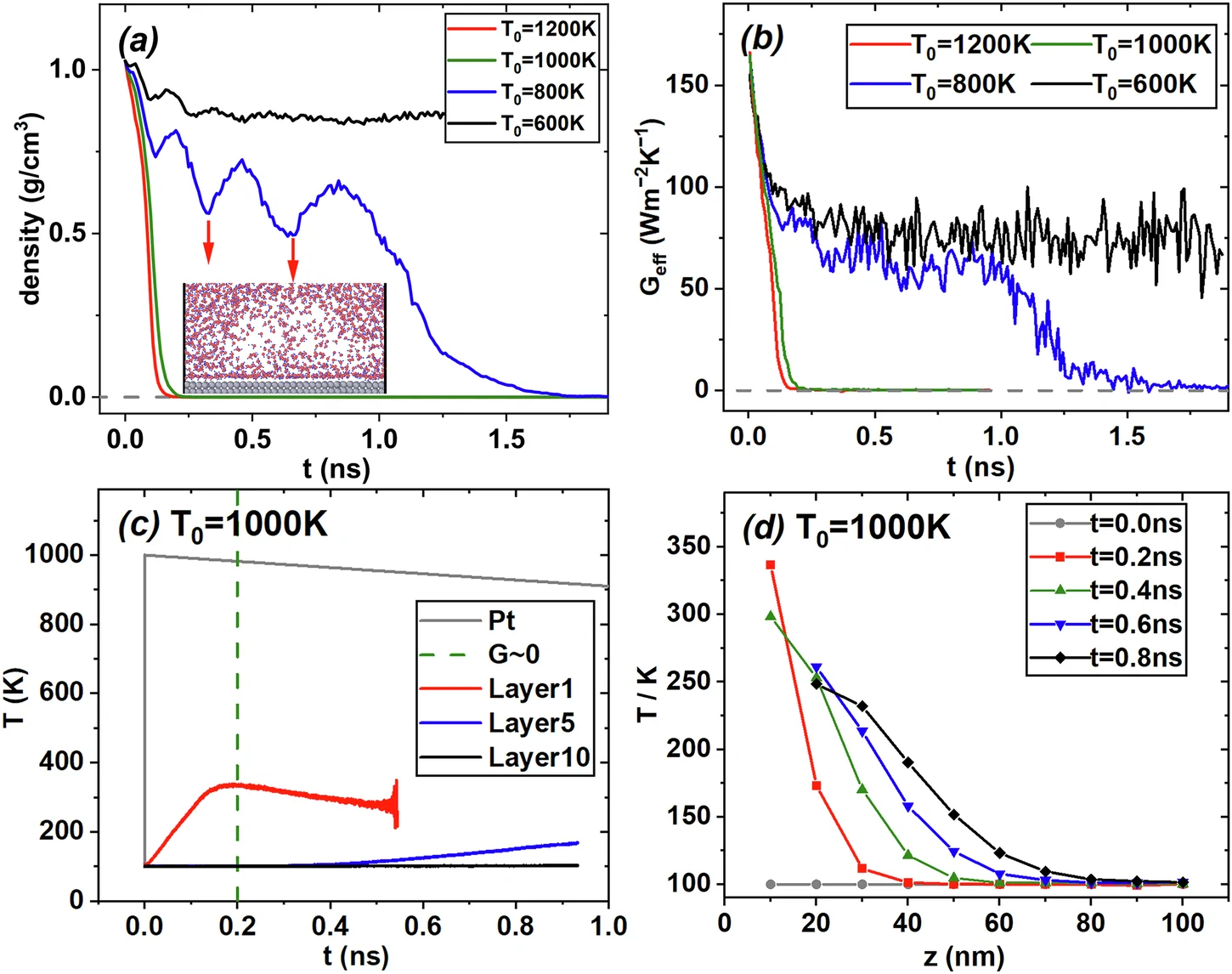
Interfacial cavity formation and its impact on heat transfer across the Pt–ASW interface. **a** Time evolution of the interfacial density ρ(t) within the first 2 nm of the ASW layer for different initial Pt temperatures. At sufficiently high temperatures (1000 and 1200 K), the interfacial density rapidly decreases to nearly zero, indicating the formation of a low-density interfacial gap (cavity), while at lower temperatures the interface remains intact. For ${T}_{0}=800 \, K$, the transient density minima are associated with the appearance of small vapour cavities at the Pt–ASW interface, as indicated by the arrows and representative configurations in (**a**). **b** Corresponding evolution of the effective interfacial thermal conductance ${G}_{{eff}}(t)$. A sharp drop in ${G}_{{eff}}(t)$ is observed concomitantly with the depletion of interfacial density, demonstrating a strong reduction of thermal coupling upon cavity formation. **c** Time evolution of temperatures in selected ASW layers (first, fifth, and tenth layers). The interfacial layer is initially heated by the Pt substrate, but its temperature exhibits a turnover and ceases to increase once the cavity forms, while deeper layers continue to heat more slowly, indicating suppressed heat transfer into the bulk ASW. After ∼0.55 ns, the particle number in the first layer becomes very low due to cavity growth, and the corresponding temperature becomes ill-defined and exhibits large fluctuations. **d** Temperature profiles T(z) at representative times before, during, and after cavity nucleation for ${T}_{0}=1000{K}$.

To further quantify the impact of vapour formation on heat transfer, we return to a thicker (100 nm) ASW model without an external thermostat, where the heat flux into the film can be directly inferred from the total energy change of the ASW. We performed simulations with initial Pt temperatures of 600, 800, 1000, and 1200 K, followed by relaxation to 100 K over 10 ns, consistent with the experimental conditions (Fig. 6). At each temperature, 3 independent simulations starting from different initial configurations were performed and yielded consistent behaviour; for clarity, only one representative trajectory is shown here. As shown in Fig. 6a, the interfacial density, ρ(t), measured within the first 2 nm of the ASW layer, exhibits a strong temperature dependence. For *T*_0_ = 1000 and 1200 K, the interfacial density rapidly drops to nearly zero at *t* ∼ 0.2 ns, indicating the formation of an interfacial vapour layer. For ${T}_{0}=800K$, the interfacial density decreases more gradually and approaches zero at later times ~2 ns, whereas for ${T}_{0}=600K$ the interface remains dense throughout the simulation. At ${T}_{0}=800K$, the interfacial density also exhibits pronounced oscillations during the early stage of heating. The corresponding configurations reveal the transient appearance of small vapour cavities near the Pt–ASW interface at the density minima around *t* = 0.32 and 0.65 ns, as shown in the insets of Fig. 6a. These observations suggest that the density oscillations originate from the formation and evolution of nanoscale vapour cavities before a continuous and stable low-density interfacial layer is established.

The formation of a nanometric vapour layer strongly suppresses heat transfer across the interface. As shown in Fig. 6b, the effective interfacial thermal conductance ${G}_{{eff}}(t)$ closely follows the evolution of the interfacial density. For *T*_0_ = 1000 and 1200 K, ${G}_{{eff}}(t)$ drops by nearly 3-4 orders of magnitude and approaches zero shortly after vapour layer formation, indicating a near-complete thermal decoupling between the Pt substrate and the ASW film. A similar but delayed reduction is observed for *T*_0_ = 800 K, while for *T*_0_ = 600 K the thermal conductance remains high. Qualitatively similar behaviour is also observed in simulations using the SPC/E water model (Figure S13).

The resulting redistribution of thermal energy within the ASW is illustrated in Fig. 6c for the representative case *T*_0_ = 1000 K. The interfacial ASW layer (layer 1) is initially heated by the Pt substrate, but its temperature exhibits a clear turnover at *t* ∼ 0.2 ns, coinciding with vapour formation. This turnover indicates that the net heat flux into the first layer becomes smaller than the heat flux it transfers to deeper layers, consistent with a strong reduction of interfacial heat input. In contrast, deeper layers (e.g., layer 5) continue to heat up gradually, while even deeper regions (layer 10) remain nearly unaffected on these timescales.

The spatial evolution of the temperature field is further shown in Fig. 6d. Prior to vapour formation, the temperature profile reflects active heat transfer from the Pt substrate into the ASW. After the cavity nucleates, however, the near-interface temperature gradient becomes significantly steeper, while the temperature rise in the bulk is strongly suppressed. This behaviour indicates that the formation of the low-density interfacial layer effectively blocks further heat input from the substrate, and the subsequent temperature evolution in the ASW is dominated by internal redistribution of energy rather than continued heating from the Pt.

We note that, although heat transfer from the Pt substrate to the adjacent ASW occurs at early times prior to cavity formation, the resulting structural response is weak, transient, and sensitive to the initial interfacial contact. In the simulations, this early-stage heating is accompanied by a small shift of the first diffraction peak (on the order of ~2%), indicating a modest local structural relaxation near the interface (see Figure S14). Consequently, such signals are not resolved in Fig. 1, which reflects an average over many pump–probe cycles. The small magnitude of the effect, combined with its short timescale and spatial localization, makes it highly susceptible to shot-to-shot fluctuations and therefore difficult to detect after averaging. The averaged response is instead dominated by the later-stage dynamics, where the formation of the interfacial vapour layer suppresses further heat input into the ASW. Once the low-density interfacial layer forms, the associated rapid decrease in ${G}_{{eff}}(t)$ strongly limits further heat transfer into the ASW. Consistent with this interpretation, the continuum calculation incorporating the MD-derived ${G}_{{eff}}(t)$ predicts that the ASW remains close to 133 K (Figure S15). This limited temperature increase is qualitatively consistent with the very small shift of the first diffraction peak (~0.05%) observed experimentally in Fig. 1.

These results provide a microscopic framework for interpreting the experimental observations. In the simulations, once the Pt surface temperature approaches ~1000 K, a nanometric interfacial vapour layer develops within nanoseconds. In the simulations, the formation of this interfacial low-density layer strongly suppresses heat transfer across the Pt–ASW boundary, corresponding to a pronounced reduction of the effective interfacial thermal conductance.

## Discussion

We investigated amorphous ice films of several hundred nm thickness, formed by vapour deposition on a Pt substrate. Note, compared to other ASW studies in literature^28^, the substrate used here is not a single crystal, but rather prepared as 30 nm thick layer on a Si wafer, which is connected to a cold finger and kept at 100 K. In summary, our optical pump – X-ray probe experiments show that the ASW film, which is heated on nanosecond timescales, exhibits minimal structural response even as the Pt substrate is impulsively heated to 700–1200 K for a few ns. This observation is inconsistent with other laser heating experiments on ASW^30–34^ and predictions from conventional Fourier heat-diffusion models^59^.

The grazing-incidence X-ray scattering geometry used here in the experiment enables two-dimensional characterization of the ASW film. A clear anisotropy is observed in the scattering pattern. The out-of-plane intensity exhibits a pronounced double peak, whereas the in-plane scattering remains single-peaked. Such anisotropic splitting is consistent with interference arising from a nanometric density modulation normal to the interface, as expected for the formation of a planar interfacial gap that partially decouples the ASW film from the underlying Pt. Complementary molecular dynamics (MD) simulations, using two different water models, support this mechanism. Under nanosecond thermal excitation, the simulations show that contact between hot Pt and ASW can rapidly generate a nanometric low-density layer at the interface within 2 ns. The resulting interfacial gap suppresses thermal contact and produces periodic modulations in the out-of-plane scattering intensity $I({q}_{z})$. The peak-to-peak spacing $\Delta q_z\propto \pi / {d}$ directly reflects the thickness *d* of the interfacial layer.

The spontaneous formation of a nanometric vapour layer provides a self-consistent microscopic mechanism for the anomalously large and pump-dependent thermal resistance observed here. Under extreme non-equilibrium conditions, interfacial heat transfer is therefore governed not by a static conductance but by the evolving structural morphology of the boundary itself. Previous studies on liquid water have shown that rapid energy deposition at solid–water interfaces can generate vapour formation, strong hydrodynamic flow, and even mechanical ejection of the liquid layer^60,61^. These responses are enabled by the high mobility of the liquid phase, where inertia and fluid motion dominate the interfacial dynamics. In contrast, the water studied here remains glassy or highly viscous, strongly suppressing hydrodynamic flow. Vapour formation is therefore confined to the interface, producing a localized structural decoupling that manifests as anisotropic scattering and strongly suppressed heat transfer. More broadly, this vapour-film–mediated decoupling suggests a potential route for dynamically limiting heat flow across interfaces driven far from equilibrium. Materials capable of rapid interfacial phase change may thus provide a transient thermal barrier under nanosecond thermal shocks.

The appearance of a transient insulation layer might remind on the Leidenfrost effect, a well-established phenomenon at macroscopic scales but poorly understood at nanometric length scales and under ultrafast heating conditions^62,63^. More broadly, the vapour-film–mediated decoupling identified here may reflect a more general non-equilibrium interfacial response. Under rapid heating, materials with relatively low vaporisation thresholds and weak interfacial coupling could undergo transient interfacial phase separation, leading to the formation of a nanometric vapour (or vacuum-like) layer and a corresponding suppression of heat transfer. Similar vapour-layer formation and the associated suppression of heat transfer have been reported in molecular dynamics studies of nanoscale boiling in simple fluids such as liquid argon, indicating that such behaviour can emerge from interfacial phase separation even in the absence of intramolecular degrees of freedom^63–66^. While this picture is based on the present ASW–Pt system, it suggests that similar interfacial decoupling may arise in other weakly bound, low-density molecular systems, or soft-matter systems, such as complex liquids or low-Z polymers under comparable ultrafast heating conditions.

## Methods

### Sample preparation and pump–probe experiments

Amorphous solid water (ASW) films were prepared via vapour deposition of ultrapure H_2_O onto a platinum surface held at 100 K under high vacuum (Figure S18). The deposition rate was controlled by adjusting the water vapour pressure through a needle valve. We determine the deposition rate by counting Newton rings visible in an optical camera image during prolonged deposition. Our sample was then deposited on a clean substrate for 4 minutes at a calculated deposition rate of 25 nm/min for a final ASW thickness of approximately 100 nm. Due to background deposition during cooling of the cryostat (small crystalline contamination—see Figure S16), samples could not be made thinner, since then such background contamination became too dominant, and the amorphous scattering signal became marginal.

The sample substrates were fabricated from standard 2-inch Si wafers with a thickness of 280 µm. The top, sample-facing, surface consisted of a sputtered 30 nm film of polycrystalline Pt. The thickness of the Pt film was confirmed with X-ray reflectivity (XRR) measurements (Figure S17). Finite element simulations (similar to the Fourier heat diffusion model presented here) indicated that a direct metal–Si interface would give instant, sub-ns timescale, heat dissipation. To slow down the thermal response, an insulating layer of SiO_2_ was introduced by baking the Si wafers at 1000 °C for over 1 h. This process yielded an oxide layer of 70–100~nm, as characterized by XRR measurements (Figure S17). A thin (~5 nm) Cr buffer layer was necessary to adhere the Pt film to SiO_2_/Si substrate. The final sample stack consisted of 100 nm ASW/30 nm Pt/5 nm Cr/70–100 nm SiO_2_/280 µm Si. The sample wafers were thermally connected to a liquid nitrogen cooled copper plate with thermal paste and retaining leaf springs (see Figure S18 for more details).

We examined the structural response of amorphous solid water (ASW) under ultrafast heating using time-resolved wide-angle X-ray scattering (WAXS) at the FXE instrument of the European XFEL^46^. A ~ 100 nm film of low-density ASW was vapour-deposited onto a Pt substrate held at 100 K under vacuum. Pump–probe experiments were carried out with an optical pump/X-ray probe configuration. The sample was excited by 800 nm optical pulses (chirped to a pulse duration of 0.6 ps) from the FXE pump–probe laser (PPL) system, which is based on a non-collinear optical parametric amplifier (NOPA). The laser operated at a repetition rate of 5 Hz and was synchronized to the X-ray pulse trains. The experiments were conducted at EuXFEL rather than using a storage-ring-based X-ray source, in order to probe the assumed short-lived states at previously unknown timescales, allowing for single-shot measurements on a weakly scattering water sample.

X-ray probe pulses (50 fs) with a photon energy of 9.3 keV (λ = 0.133 nm) were delivered at 10 Hz repetition rate in a grazing-incidence geometry, providing a momentum-transfer range of 0.5–3.5 Å^−1^. Pump–probe delays up to 10 ns were realized by adjusting the relative timing between the optical and X-ray pulses. The X-ray beam was focused to 10 × 10 µm^2^ FWHM, and the pump laser focus was ~80 ×80 µm^2^ FWHM, providing a homogeneously pumped area for probing. In grazing incidence, the footprints are elongated along the scattering plane by a factor of 190, for X-rays at 0.3 degrees incidence, and a factor of 40 for pump laser at 1.3 degrees incidence. During each measurement, the pump pulse transiently heated the Pt film, and the corresponding Pt temperature evolution was extracted from the shift of its (111) and (200) diffraction peaks in the WAXS patterns. The structural response of the ASW layer was monitored from the time-dependent WAXS profiles of its first diffraction peak.

The raw detector data was converted into analyzable pump–probe diffraction data by azimuthal integration and grouped averaging. Integration from the 2D pixel domain to 1D I(q) curves was carried out using the standard PyFAI Python library^67^.These integrated diffraction patterns were then sorted into pumped and unpumped shots (5 Hz pump, 10 Hz probe), and grouped by delay time (for delay scans) or pump fluence (for fluence scans). Data was accumulated for 5–30 s at each scan step, corresponding to 50–300 X-ray pulses (half pumped, half unpumped). Equivalent data was averaged together, giving a series of low-noise diffraction patterns of a pumped state and an unpumped state for each scan step in the measurements.

Pump-induced temperature changes in platinum were derived from the shift in q position of the Pt Bragg reflections. A Voigt profile and linear baseline correction were fitted to each of the three relevant q-ranges (1.2–2.2 Å^−1^ for the ASW peak, 2.5–3.0 Å^−1^ for Pt (111), and 3.0–3.5 Å^−1^ for Pt (200)) for every group-averaged diffraction pattern described above. For simplicity, the Voigt parameters were constrained such that the standard deviation and scale of the two underlying distributions were set equal (i.e., $\sigma =\gamma$ in the standard notation). From the Voigt centers, we computed the pump-induced linear expansion as

$$\frac{\Delta d}{d}=\frac{{d}_{{{{\rm{pump}}}}}-{d}_{0}}{{d}_{0}}=\frac{{q}_{0}-{q}_{{{{\rm{pump}}}}}}{{q}_{{{{\rm{pump}}}}}}$$

(the inversion $0\leftrightarrow {{{\rm{pump}}}}$ is due to the reciprocal relationship between length and scattering vector). The unpumped reference, ${q}_{0}$, was always taken from the same scan step as ${q}_{{{{\rm{pump}}}}}$ in order to cancel any long-term drift in beam position. Finally, the Pt temperature (i.e., increase from the measured substrate temperature) was interpolated from tabulated thermal expansion data^55^. The linear expansion of the two Pt Bragg reflections was consistently in very good agreement, and we therefore used the average from both peaks to compute the temperature. The experimentally determined Pt temperature evolution under different pump fluences at a fixed pump–probe delay of 1.2 ns is shown in Figure S19.

### One-dimensional heat transfer modelling

To assess whether conventional heat conduction can account for the observed thermal decoupling, we solved the one-dimensional heat equation

2.1 $${\rho C}_{{{p}}}\frac{\partial {{T}}}{\partial {t}}=\frac{\partial }{\partial {{z}}}\left[\frac{{{k}}\partial {{T}}}{\partial {{z}}}\right],$$

for a Pt–interface–ASW stack consisting of a 5-nm Pt layer, a 5-nm interfacial region, and a 100-nm ASW film. The equation was discretized on a uniform, cell-centered spatial grid (Δz = 0.5 nm) and advanced in time using an explicit finite-volume scheme. The heat flux across each cell boundary was evaluated using the harmonic mean of the thermal conductivities of the two adjacent cells. The time step was selected automatically to satisfy the stability criterion of the explicit scheme.

The Pt surface was assigned the experimentally measured time-dependent temperature ${T}_{{Pt}}(t)$, while the outer ASW surface was taken to be adiabatic. Material parameters were expressed through the thermal diffusivity $\alpha =k/(\rho {C}_{P})$. Room-temperature bulk values were used: ${C}_{P}=25.648\,{{{\rm{J\; mo}}}}{{{{\rm{l}}}}}^{-1}{{{{\rm{K}}}}}^{-1}=132\,{{{\rm{J}}}}{{{{\rm{kg}}}}}^{-1}{{{{\rm{K}}}}}^{{-}1}$ and ${\rho }_{{Pt}}=21.45\,{g}{{cm}}^{-3}$^55^. The thermal conductivity was set to *k*_*Pt*_ = 72 *W* m^−1^ K^−1^^50^, yielding a thermal diffusivity of *α*_*Pt*_= 2.5×10^−5^ m^2^ s^−1^.

The ASW film corresponds structurally and thermodynamically to low-density amorphous ice (LDA). Its density in the 77–100 K range is ${\rho }_{{ASW}}=0.94{g\; c}{m}^{-3}$^54^. The low-temperature heat capacity of amorphous ice is ${C}_{P}=\,15{{{\rm{J\; mo}}}}{{{{\rm{l}}}}}^{-1}{{{{\rm{K}}}}}^{-1}=830 \, {{{\rm{J}}}}{{{{\rm{kg}}}}}^{-1}{{{{\rm{K}}}}}^{{-}1}$^56^, and Andersson and Suga report a thermal conductivity of *k*_*ASW*_ = 0.6 W m^−1^ K^−1^ for amorphous ice in the 70–135 K range^51,52,57^, which, together with the density and heat-capacity values above, corresponds to a thermal diffusivity of *α*_*ASW*_ = 7.7 × 10^−7^ m^2^ s^−1^.

Heat transfer across the Pt–ASW interface is characterized by a Kapitza conductance *G*: $J={G}\Delta T$. Based on literature values from both experiments and molecular-dynamics simulations, we adopted a representative interfacial thermal conductance of *G* ≈ 130 MW m^−2^ K^−1^ for the Pt–ASW interface^53^. Given the partial wettability of water on Pt (Figure S5), this conductance provides a physically reasonable estimate for our system. In the numerical implementation, the interfacial resistance was represented by assigning the 5-nm effective interfacial region a thermal conductivity *k*_*int*_ = G·*L*_*int*_ ≈ 0.65 W m^−1^ K^−1^, such that its steady-state areal thermal resistance is equal to (1/G).

### Molecular dynamics simulations

We performed molecular dynamics (MD) simulations of ultrafast heating in Pt–ASW slabs with initially ideal interfacial contact. In the main text, water was modelled using the TIP4P/2005 potential^48^, with Lennard-Jones interactions acting on oxygen and partial charges distributed on the molecular sites, while molecular rigidity was enforced via SHAKE^49,68^. Long-range Coulomb interactions were treated using PPPM under three-dimensional periodic boundary conditions^69^. Platinum atoms interacted through a Lennard-Jones potential, and Pt–water cross interactions followed Lorentz–Berthelot mixing; no explicit interfacial resistance was imposed. The Pt–O interaction strength was chosen such that the Pt surface remained effectively neutral—neither strongly hydrophilic nor hydrophobic—consistent with an equilibrium contact angle of approximately 90°. To assess the robustness of the results, additional simulations using the SPC/E water model^49^ were performed, which yielded qualitatively consistent behaviour (see Supplementary Information).

In the MD simulations, we modelled a 100-nm ASW slab supported on a 2-nm Pt substrate. The system was first equilibrated and then rapidly quenched from 350 K to 100 K to generate amorphous solid water prior to the heating runs. In the pump–probe stage, only the Pt slab was driven by the experimental temperature history T(t), while the ASW evolved in the microcanonical (NVE) ensemble to capture its non-equilibrium thermal response. During simulations, the structural and thermodynamic responses of ASW were characterized by temperature and density profiles T(z,t) and ρ(z,t), radial distribution functions g(r), and structure factors ${{{\rm{S}}}}(q)$. The ASW temperature profiles obtained from continuum heat-conduction calculations and corresponding SPC/E molecular dynamics simulations under Pt heating–cooling conditions are compared in Figure S20.

### Direction-resolved structure factor

To quantify the anisotropic structural response of the ASW film, we computed the static structure factor directly from its microscopic definition,

$${{S}}({{{\boldsymbol{q}}}})=\frac{1}{N}\left\langle {\rho }_{{{{\boldsymbol{q}}}}}\,{\rho }_{-{{{\boldsymbol{q}}}}}\right\rangle ,{\rho }_{{{{\boldsymbol{q}}}}}={\sum }_{j=1}^{N}{e}^{i{{{\boldsymbol{q}}}}{{{\rm{\cdot }}}}{{{{\boldsymbol{r}}}}}_{{{{\boldsymbol{j}}}}}},$$

using the oxygen atoms. For each saved configuration, atomic positions were wrapped back into the simulation cell to remove artifacts from periodic boundaries. Because the pump excitation and subsequent heating impose a strong structural anisotropy along the film normal, we did not perform an isotropic spherical average. Instead, we evaluated ${{S}}({{{\boldsymbol{q}}}})$ by averaging over two well-defined ensembles of wavevectors. Hereafter, ${{S}}({q}_{xy})$ and ${{S}}({q}_{z})$ denote averages over wavevectors oriented predominantly within the (*xy*) plane and along the (*z*) direction, respectively, at the same magnitude (*q*).:

In-plane (*xy*) directions:

We generated random unit vectors $\hat{{{{\boldsymbol{q}}}}}$ whose in-plane projection satisfies $\sqrt{{\hat{{q}_{x}}}^{2}+\left.{\hat{{q}_{y}}}^{2}\right)}\,\ge \,0.8$, ensuring that $\hat{{{{\boldsymbol{q}}}}}$ lies predominantly within the xy plane. For each magnitude q, we set ${{{\boldsymbol{q}}}}=q\hat{{{{\boldsymbol{q}}}}}$ and computed ${{S}}({{{\boldsymbol{q}}}})$; the in-plane structure factor was then obtained as ${S}$(${q_{xy}}$)= ${\left\langle {{S}}({{{\boldsymbol{q}}}})\right\rangle }_{{xy}}$

Out-of-plane (*z*) directions:

To resolve structural correlations along the film normal, we generated random unit vectors satisfying $\hat{{q}_{z}}$ $\ge \,0.8$, ensuring that *q* is strongly oriented along ±z. The corresponding average, ${S}$(${q_z}$)= ${\left\langle {{S}}({{{\boldsymbol{q}}}})\right\rangle }_{z}$ isolates density correlations along z direction.

This directional sampling gives two structure-factor components$\,{S}$(${q_{xy}}$) and ${S}$(${q_{z}}$) both derived from the same microscopic definition but probing orthogonal structural sectors of reciprocal space. The magnitude of the scattering vector was scanned uniformly from ${q}=\,1-\,4\,{{{{\AA }}}}^{-1}$ using 500 discrete values. The reported curves were obtained by averaging the directional ${{S}}(q)$ over 200 MD configurations over 4 ns. Representative TIP4P/2005 and SPC/E configurations with different vapour-gap thicknesses, used for the corresponding structure-factor calculations, are shown in Figure S10 and S21.

### 1D model demonstrating oscillations in ${{{\boldsymbol{S}}}}({{{{\boldsymbol{q}}}}}_{{{{\boldsymbol{Z}}}}})$

Direction-resolved pair distribution functions (Figure S11) show that the first- and second-neighbour shells shift anisotropically when a cavity is introduced. These changes account for the difference between the peak positions of ${{S}}({q}_{z})$ and ${{S}}({q}_{{xy}})$, reflecting an anisotropic local packing near the interface. However, the pair correlations remain smooth and single-peaked in both directions and therefore do not generate the double-peak structure in ${{S}}({q}_{z})$. This indicates that the peak splitting originates not from a modification of the local coordination shell but from the long-wavelength non-uniformity of the density profile introduced by the vapour gap. To illustrate why a nanometric vacuum layer embedded inside an otherwise dense amorphous film necessarily produces an oscillatory modulation in the out-of-plane structure factor, we consider a minimal one-dimensional model. The density profile is taken as two semi-infinite regions of constant density ${\rho }_{0}$ separated by a vacuum gap of width d:

$$\rho (z) = \left\{\begin{array}{l}{\rho}_{0}, \quad z < 0 \\ 0, 0 < z < d \\ {\rho }_{0}, \quad z > d\end{array}\right.$$

Under the kinematic approximation, the scattering amplitude is the Fourier transform of the density profile:

$$\,A\left({q}_{z}\right)\propto \,\int \rho \left(z\right){e}^{-i{q}_{z}\cdot z}{dz}={A}_{1}\left({q}_{z}\right)+{A}_{2}\left({q}_{z}\right)$$

For the experimentally relevant range ${q}_{z} > 0$, the two contributions are

$$A_{1}(q_z)=\rho_0\int_{-\infty}^{0}e^{-iq_zz}\,dz=\frac{\rho_0}{-iq_z}$$

$$A_{2}\left(q_{z}\right)=\rho_{0}\int_{d}^{\infty}e^{-iq_{z}z}\,dz=\frac{\rho_{0}e^{-iq_{z}d}}{iq_{z}}$$

$$A\left({q}_{z}\right)\propto {A}_{1}\left({q}_{z}\right)+{A}_{2}\left({q}_{z}\right)={\rho }_{0}\frac{{1-e}^{-i{q}_{z}\cdot d}}{-{i{q}_{z}}}$$

The out-of-plane structure factor is proportional to the modulus square of the Fourier amplitude of the density profile, ${{S}}({q_z})\propto {{|A}({q_z})|}^{2}$. This yields

$$S(q_z)\propto\frac{2\rho_0^2}{q_z^2}\left[1-\cos(q_zd)\right]$$

producing an interference modulation with a full period $\Delta {q}_{z} \sim 2\pi /d$.

### Calculating interfacial thermal conductance

The effective time-dependent interfacial thermal conductance ${G}_{{eff}}(t)$ across the Pt–ASW interface was evaluated from non-equilibrium molecular dynamics (MD) simulations performed using LAMMPS. The 100-nm ASW film was uniformly divided into ten layers, each with a thickness of 10 nm. During the simulations, the kinetic energy, potential energy, and temperature of each ASW layer were computed and recorded every 100 timesteps. The interfacial heat flux density normal to the surface was obtained as $J(t)=\frac{1}{A}\frac{d{E}_{{ASW}}}{{dt}}$, where ${E}_{{ASW}}$ is the total internal energy of the ASW film and A is the Pt–ASW interfacial area. The interfacial temperature difference was determined from the temperatures of the Pt substrate and the first ASW layer, $\Delta T(t)={T}_{{Pt}}-{T}_{{ASW},1}$. Finally, the effective time-dependent interfacial thermal conductance was obtained from ${G}_{{eff}}(t)=\frac{{{{\rm{J}}}}({{{\rm{t}}}})}{\Delta T(t)}$. Here, ${G}_{{eff}}(t)$ denotes the MD-derived effective conductance and is distinguished from the constant reference conductance, *G*=130 MW m^−2^ K^−1^, used in the conventional continuum heat-conduction calculation.

## Supplementary information


Supplemental information


## Data Availability

X-ray data were recorded May 2023 at the European XFEL, are stored at the facility and are available under^70^. Processed data and codes that support the findings of this study are available under https://doi.org/10.5281/zenodo.21250189^71^.

## Code availability

The custom code used for the continuum heat-transfer calculations and for calculating $S({q}_{{xy}})$ and $S({q}_{z})$ is available in a repository at https://doi.org/10.5281/zenodo.21250189^71^.
