# Supplementary material for: Formation of nanoscale vapour films governing thermal resistance in amorphous ice
Source: Commun Chem. 2026 Sep 1;9:289. doi: 10.1038/s42004-026-02177-2 (PMC13534528; doi:10.1038/s42004-026-02177-2)
Supplement: Supplementary file 1 [file 42004_2026_2177_MOESM1_ESM.pdf]

Supplementary information

to "Formation of nanoscale vapour films governing thermal resistance in amorphous ice"

Yizhi Liu[1,9], Tobias Eklund[1,2,3,9],

Aigerim Karina[4], Svenja Hövelmann[5,6], Robert PC Bauer[7], Seonju You[8], Claudia Goy[7], Nele N. Striker[7], Alexander Gierke[3,7], Kenneth Hess[1], Kai Schlage[7], Lars Bocklage[7], Fernando Ardana Lamas[3], Hao Wang[3], Yifeng Jiang[3], Yohei Uemara[3], Chris Milne[3], Kyung Hwan Kim[8], Bridget Murphy[5,6], Felix Lehmkühler[7], Peter Zalden[3], Katrin Amann-Winkel[1,2,*]

1 Max Planck Institute for Polymer Research, Ackermannweg 10, 55128 Mainz, Germany

2 Institut für Physik, Johannes Gutenberg-Universität Mainz, 55099 Mainz, Germany

3 European XFEL, Holzkoppel 4, Schenefeld 22869, Germany

4 Department of Chemical Physics, Fysikum, Stockholm University, 10691 Stockholm, Sweden

5 Institute of Experimental and Applied Physics, Kiel University, Leibnizstraße 19, 24118 Kiel, Germany

6 Ruprecht Haensel Laboratory, Deutsches Elektronen-Synchrotron DESY, Notkestraße 85, 22607 Hamburg, Germany

7 Deutsches Elektronen-Synchrotron DESY, Notkestraße 85, 22607 Hamburg, Germany

8 Department of Chemistry, POSTECH, Pohang 37673, Republic of Korea

[9]equally contributing to this work

Corresponding author:

[*]amannk@mpip-mainz.mpg.de

The Supplementary Information provides additional data and analysis supporting the results presented in the main text. Figures S1–S21 contain supplementary experimental and simulation results referenced throughout the manuscript.
In the main text, we present results obtained using the TIP4P/2005 water model. To examine the robustness of our conclusions with respect to the choice of water model, we performed **additional simulations using the SPC/E model**. The corresponding results are shown in Figures S3, S4, S8, S9, S13, S20 and S21. We find that the key interfacial behavior, including the formation of the vapor layer and the associated suppression of heat transfer, remains qualitatively unchanged, confirming that our conclusions are not sensitive to the specific water model employed.
Additional results from our **TIP4P/2005** simulation results, supporting the main text, are shown in Figures S10, S11 and S14.

The Supplementary Information contains further information about the **experimental setup** (Fig. S18) as well as the **pre-characterization** of the substrate. These are, contact-angle measurements (S5) and thickness determination using X-ray reflectivity (XRR) (S17). Figure S16 shows the X-ray scattering results from a bare Si–Pt substrate, taken prior to the experiments during cooling from 200 K to ~125 K.
Supporting experimental data from the **pump-probe experiments** are provided in Figures S1, S2, S6, S7, S12 and S19. Figure S19 presents the experimentally determined temporal evolution of the Pt temperature during fluence scans, showing the transient heating dynamics T(t) at a fixed pump–probe delay of 1.2 ns.

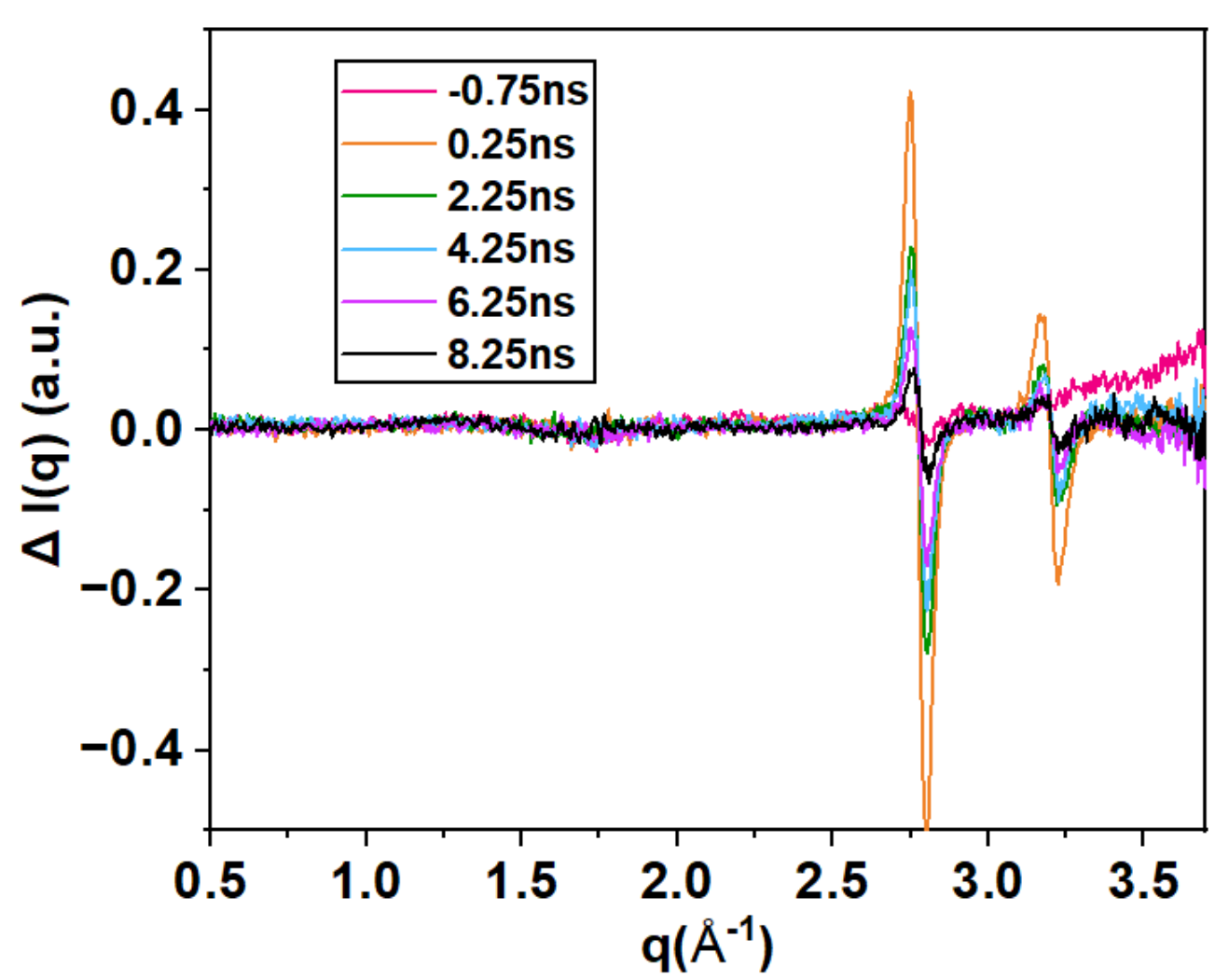

Figure S1. Differential diffraction intensity $\Delta I(q) = I_{on}(q) - I_{off}(q)$ at the same pump–probe delay times as in the main text. The data are obtained by subtracting the averaged pump-off signal from the corresponding pump-on signal at each delay, highlighting the transient structural response induced by laser excitation.

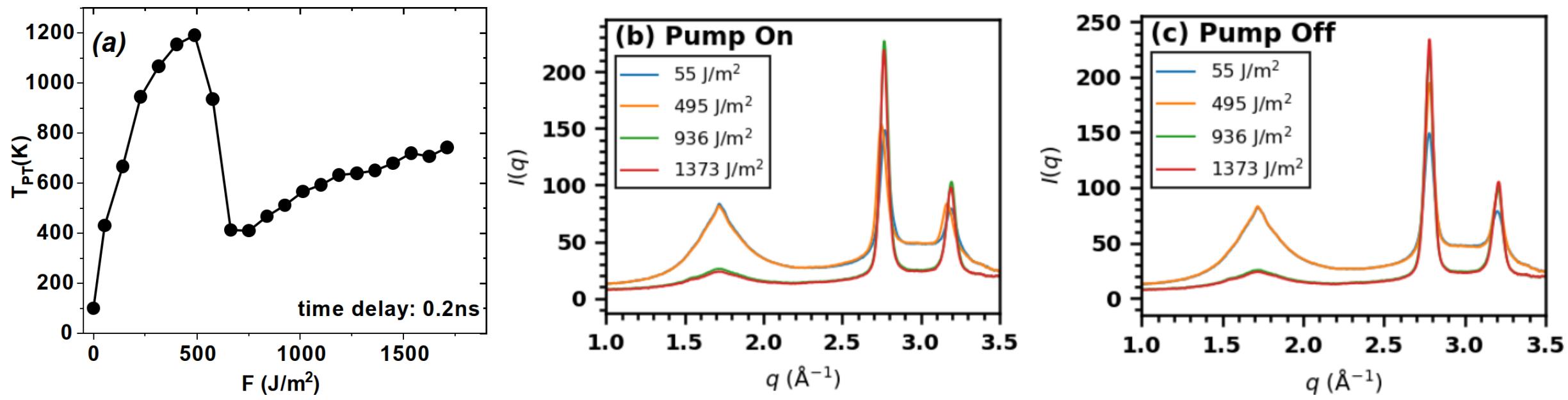

Figure S2. The **diffraction profiles** of Pt and ASW recorded at various pump–probe fluences. Pump–probe measurements on amorphous solid water (ASW) deposited on a Pt substrate and the resulting transient structural and thermal response. (a) Experimentally determined temperature of Pt at 0.25 ns after pumping with various pump laser fluences. (b) X-ray scattering intensity I(q) recorded 0.25 ns after the pump pulse for different pump fluences. (c) Reference I(q) profiles collected without applying the pump pulse. At fluences approaching ~500 J/m^2, a qualitative change in the Pt temperature is observed, marking a threshold at which the ASW layer begins to be ablated from the substrate—likely caused by partial melting or restructuring of the Pt film under extreme excitation. Each diffraction pattern is averaged from 100 X-ray pulses.

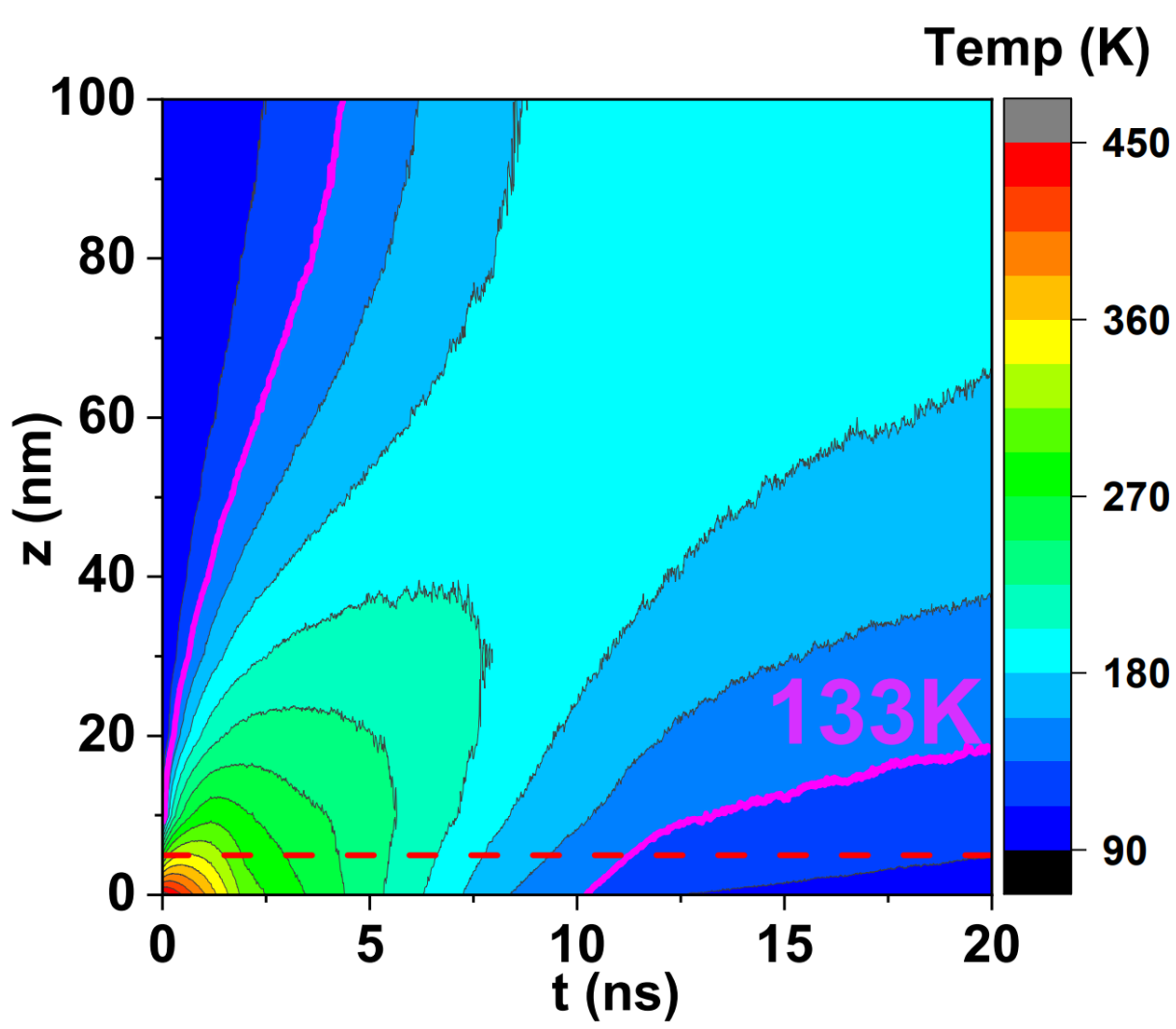

Figure S3. Temperature profiles $T(z,t)$ of the ASW film obtained from molecular dynamics simulations using the **SPC/E water model** under Pt heating. A slightly faster heating rate is observed in SPC/E, while the overall spatiotemporal characteristics remain consistent. The magenta contour denotes a reference isotherm at 133 K, used to track the propagation of the thermal front. The red dashed lines indicate the Pt–ASW interface.

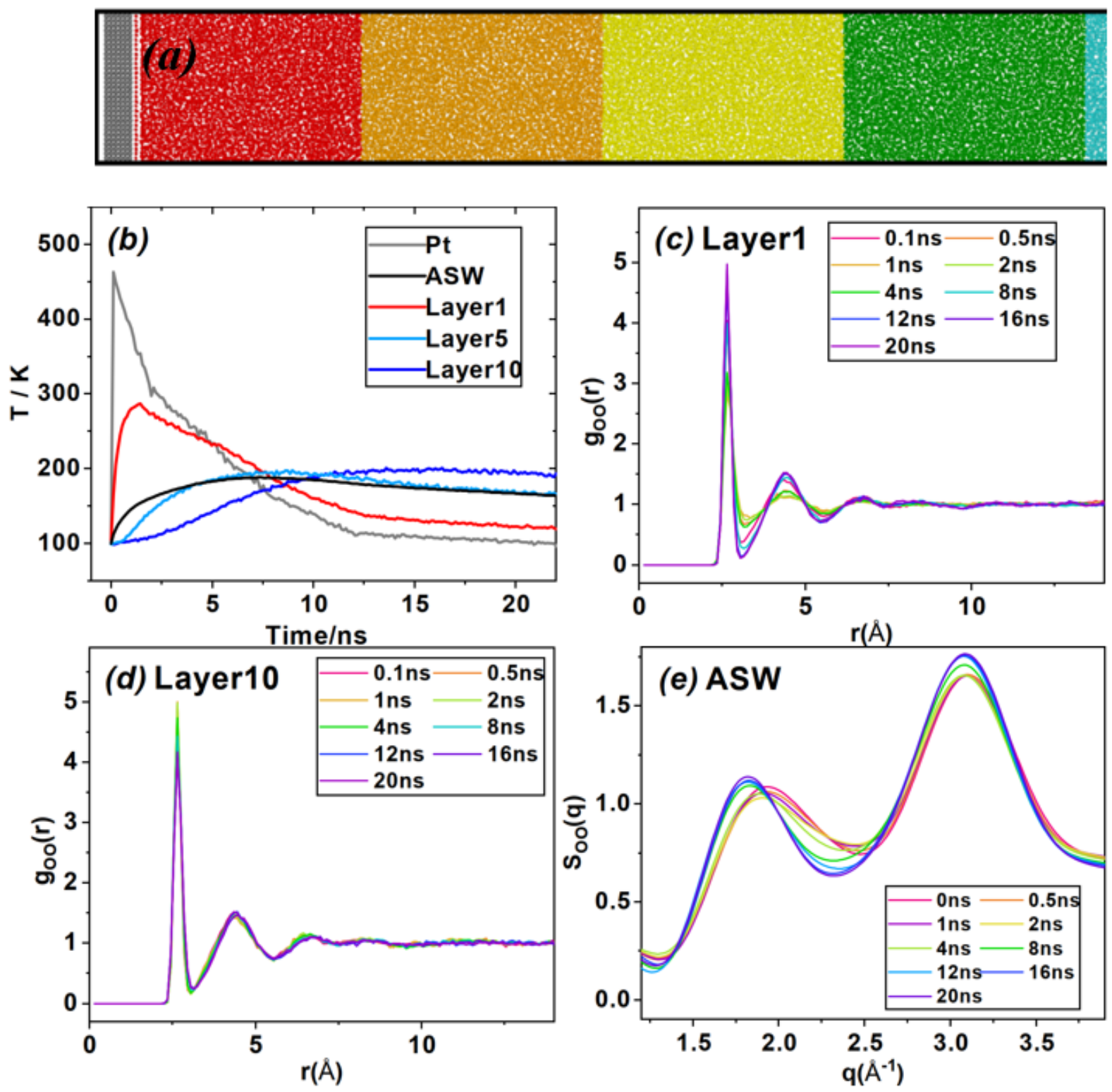

Figure S4. Layer-resolved structural evolution of ASW during MD heating **simulations using the SPC/E** model. (a) Schematic illustration of the simulation setup and division of the ASW film into layers, with the first layer (adjacent to Pt) highlighted in red. (b) Time evolution of temperature T(t) for the Pt substrate (grey), the first (red) and tenth (blue) ASW layers, and the average over the entire ASW film (black). (c,d) Layer-resolved pair distribution functions $g_{OO}(r)$ for the first (near-interface) (c) and tenth (interior) layers (d), highlighting the spatially heterogeneous structural response. (e) Total structure factor S(q) obtained from the Fourier transform of the atomic configurations, averaged over all layers.

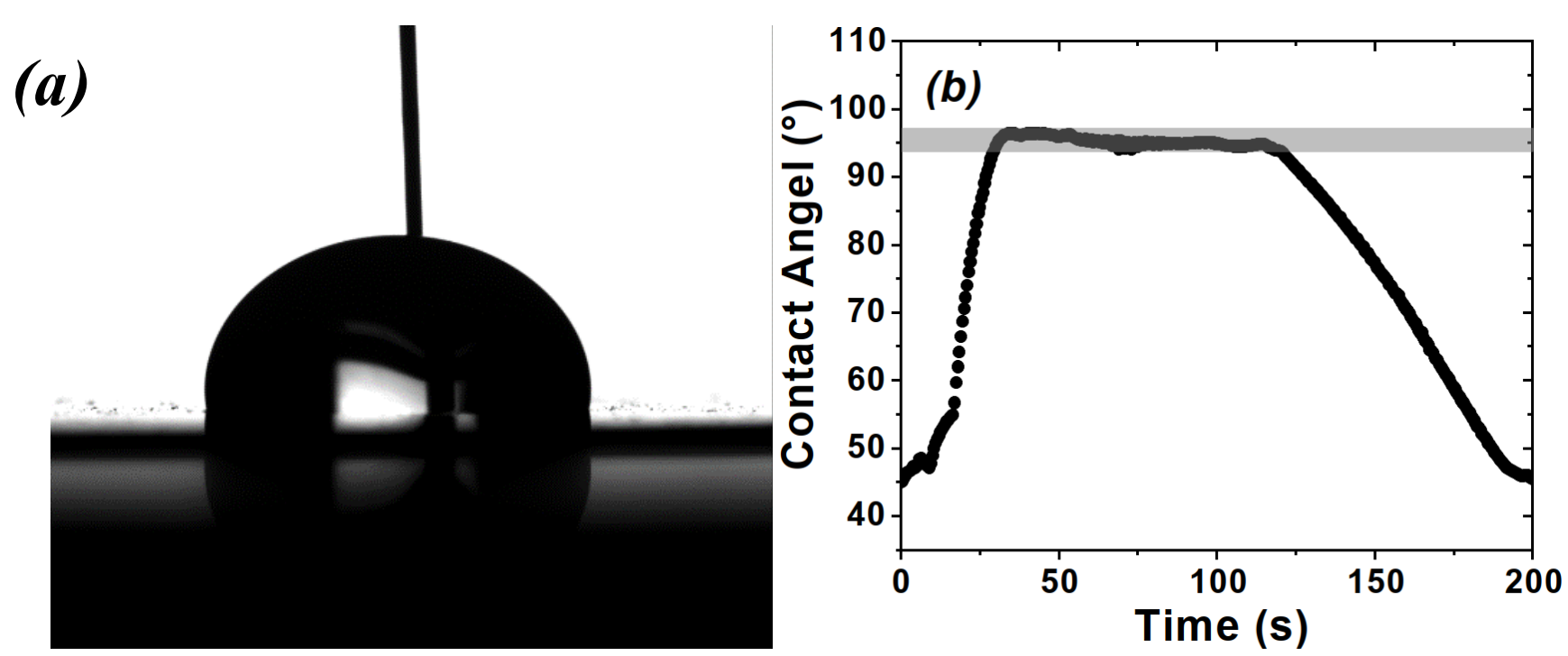

Figure S5. Wettability characterization of the Pt substrate using dynamic **contact-angle measurements**. (a) Representative snapshot of the water droplet on Pt, showing a static contact angle of ~95°. (b) Time evolution of the contact angle during cycles of water injection and withdrawal. The transient minima arise during receding stages, while a stable plateau around ~90° persists in quasi-static periods, reflecting the intrinsic wettability of the Pt surface.

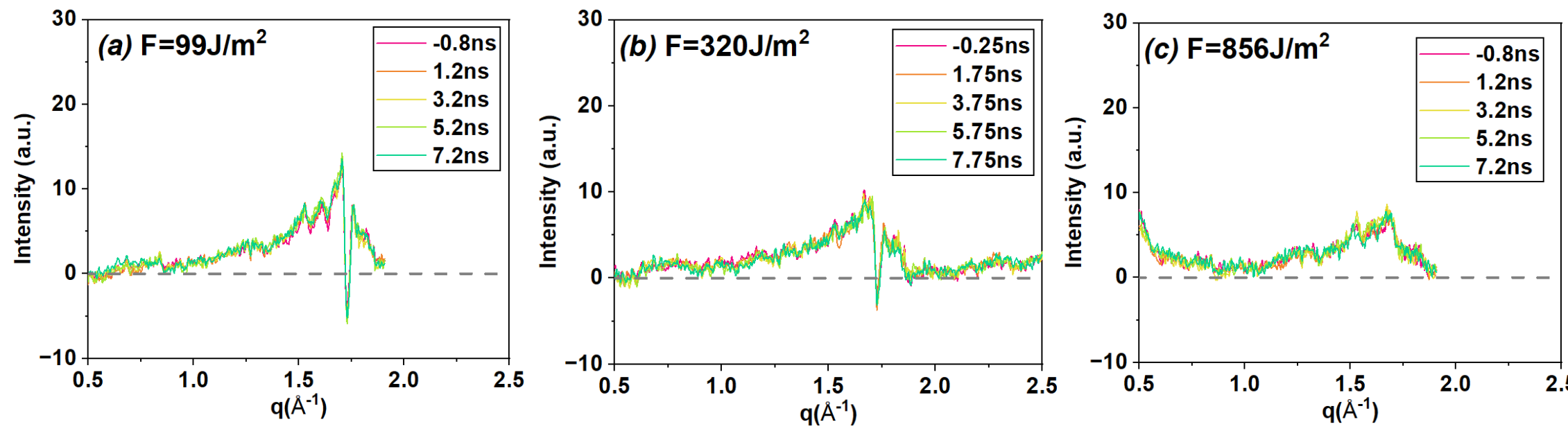

Figure S6. Anisotropic diffraction response of the ASW layer under pump–probe excitation. Difference between the in-plane and out-of-plane X-ray scattering intensities, $\Delta I(q) = I_{xy}(q) - I_z(q)$, measured at selected pump–probe delays for three pump fluences: **(a)** 99 $\mathrm{J/m^{-2}}$ (difference patterns calculated from 150 pumped X-ray pulses averaged), **(b)** 320 $\mathrm{J/m^{-2}}$ (25 pumped X-ray pulses averaged), and **(c)** 856 $\mathrm{J/m^{-2}}$ (50 pumped X-ray pulses averaged). Different colors correspond to different pump–probe delays, as indicated. Notably, the anisotropic signal $\Delta I(q)$ is already present at the earliest measured pump–probe delay and shows no discernible evolution with time. This behavior suggests that the anisotropy is not driven by gradual heating of the ASW film. Instead, it is consistent with an interfacial origin, which we associate with the formation of a vapor layer at the Pt–ASW interface upon pumping.

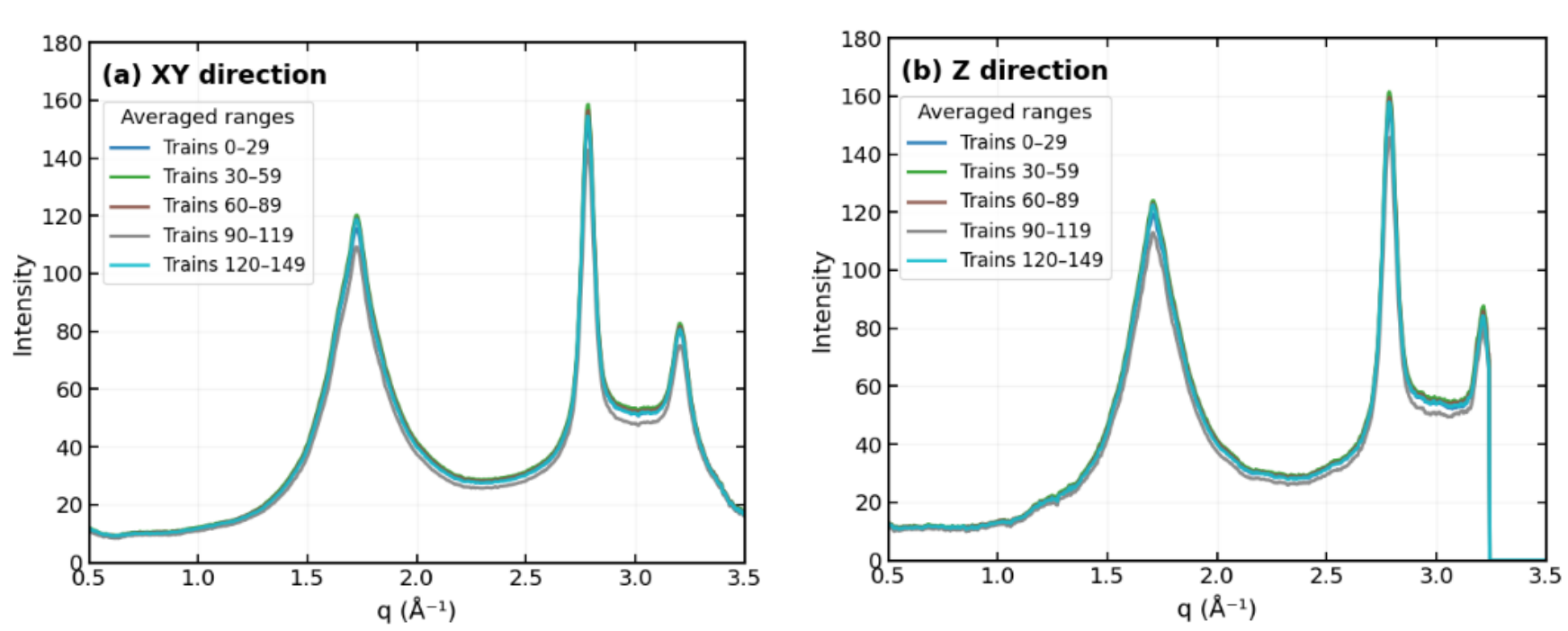

Figure S7. X-ray–only stability test of the ASW scattering signal. Average I(q) computed from consecutive X-ray probe trains in the absence of pump excitation. Each curve represents the mean of 30 trains (each train containing 30 X-ray pulses). (a) XY-direction scattering. (b) Z-direction scattering. All curves nearly overlap, demonstrating that prolonged X-ray exposure alone does not induce any detectable structural evolution or crystallization in the amorphous ice film. No double peak is observed in the Z direction.

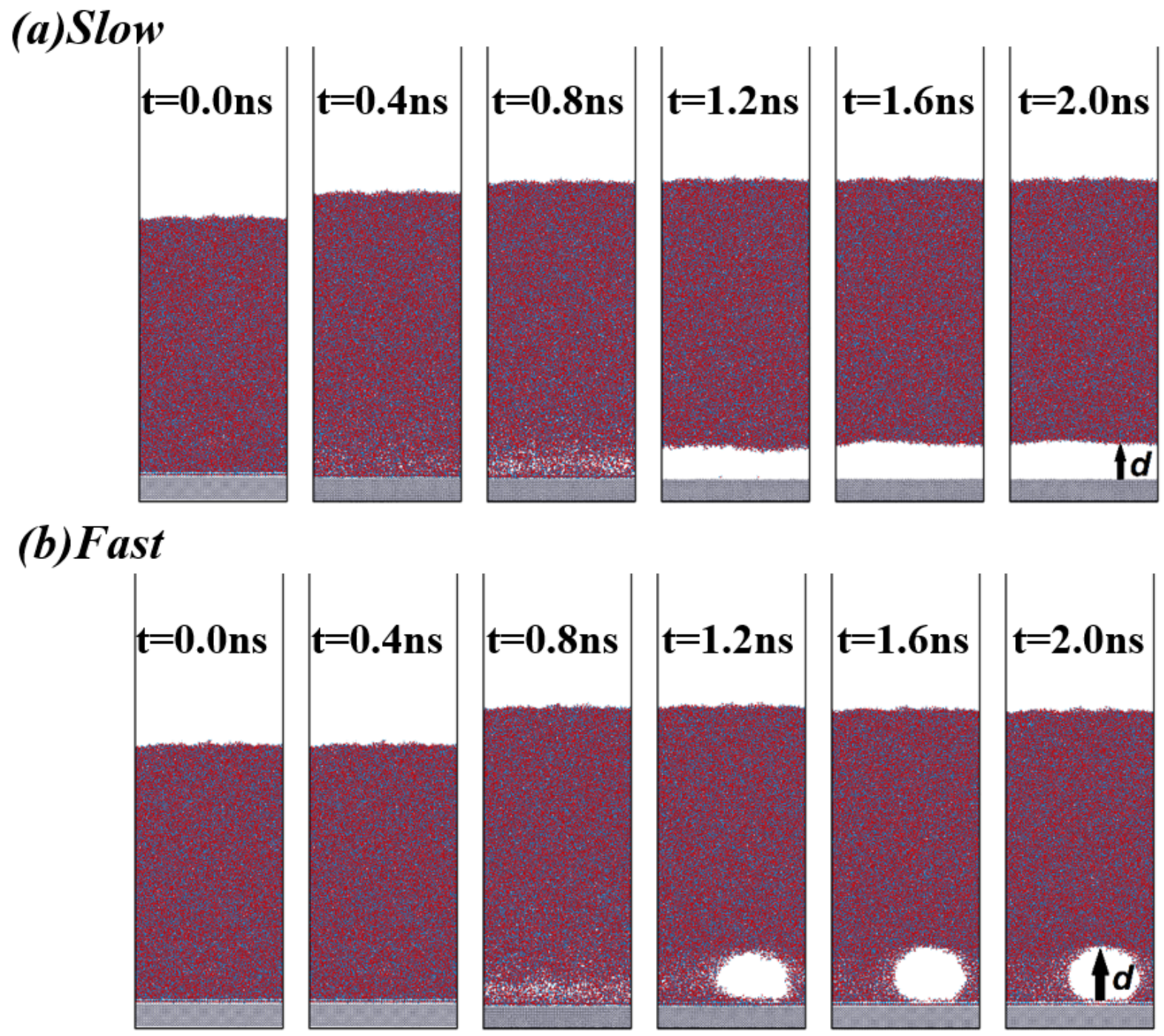

Figure S8. Molecular dynamics evidence of vapor-layer formation at the Pt–ASW interface using the **SPC/E water model**. (a, b) MD snapshots at t = 0, 0.4, 0.8, 1.2, 1.6, 2.0 ns showing the structural evolution of the Pt–ASW boundary following impulsive heating of the Pt slab to 1000 K. Two cooling protocols are shown: (a) slow quench (10 ns) and (b) fast quench (1 ns). In both cases, a nanometric low-density layer develops at the interface within ~1 ns. The arrow indicates the thickness of the low-density layer. Red and blue spheres denote oxygen and hydrogen atoms, respectively.

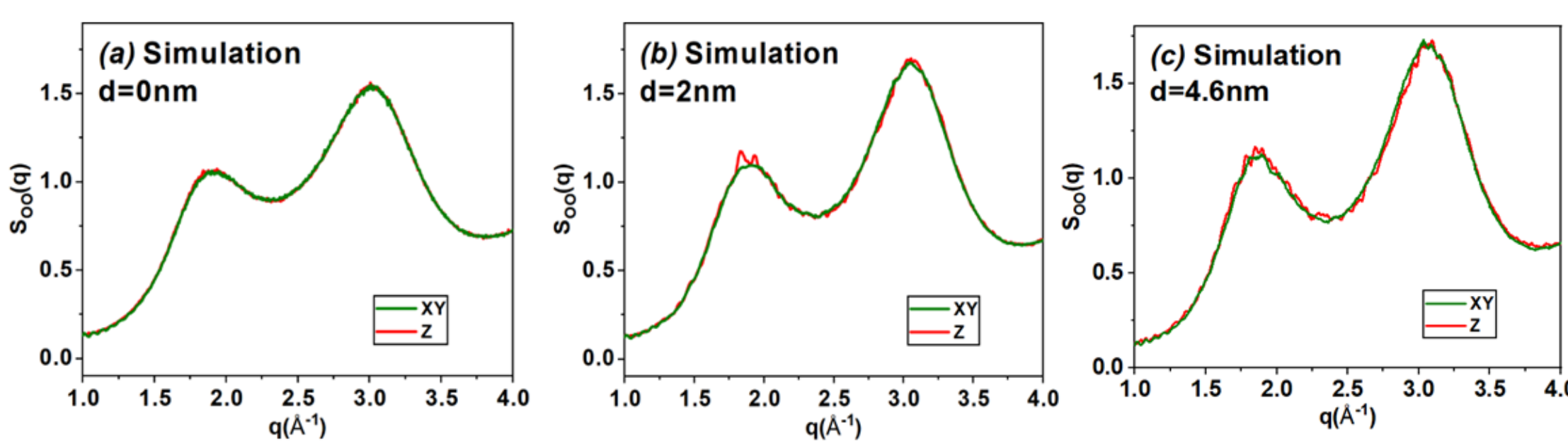

Figure S9. Scattering signatures of an interfacial vapor layer from molecular dynamics simulations using the **SPC/E water model** (configurations shown in Fig. S20). (a–c) MD-calculated scattering profiles $S(q_z)$ and $S(q_{xy})$ for three interfacial conditions: (a) no gap, (b) a 2 nm vapor gap, and (c) a 4.6 nm vapor gap. The presence of a nanometric low-density interfacial layer leads to a characteristic splitting in $S(q_z)$, which increases with gap thickness due to interference between the two interfaces. The corresponding periodicity follows $\Delta qz=\pi/d$, where d is the thickness of the interfacial layer.

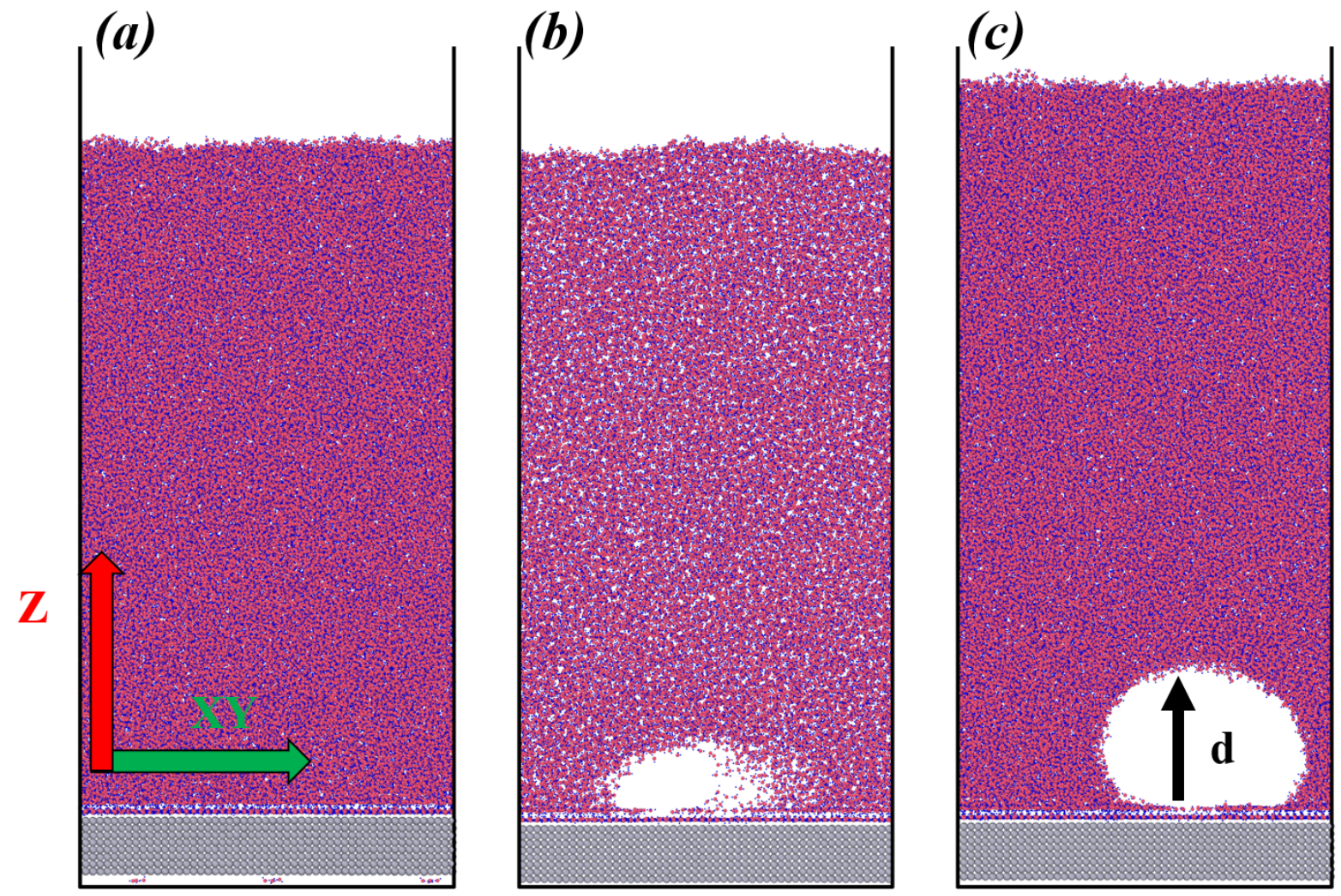

Figure S10. Representative atomic configurations with different vapor-gap thicknesses used for structure-factor calculations, obtained using the **TIP4P/2005 water model**. (a) Configuration without a vapor gap at the Pt–ASW interface, obtained by keeping the Pt slab fixed at 250 K, which suppresses the formation of the interfacial low-density layer. (b) Configuration with a ~2 nm vapor layer near the interface; only half of the simulation box along the x direction is shown for clarity, as the low-density region is located near the center of the system. (c) Configuration with a ~4.6 nm vapor layer at the interface.

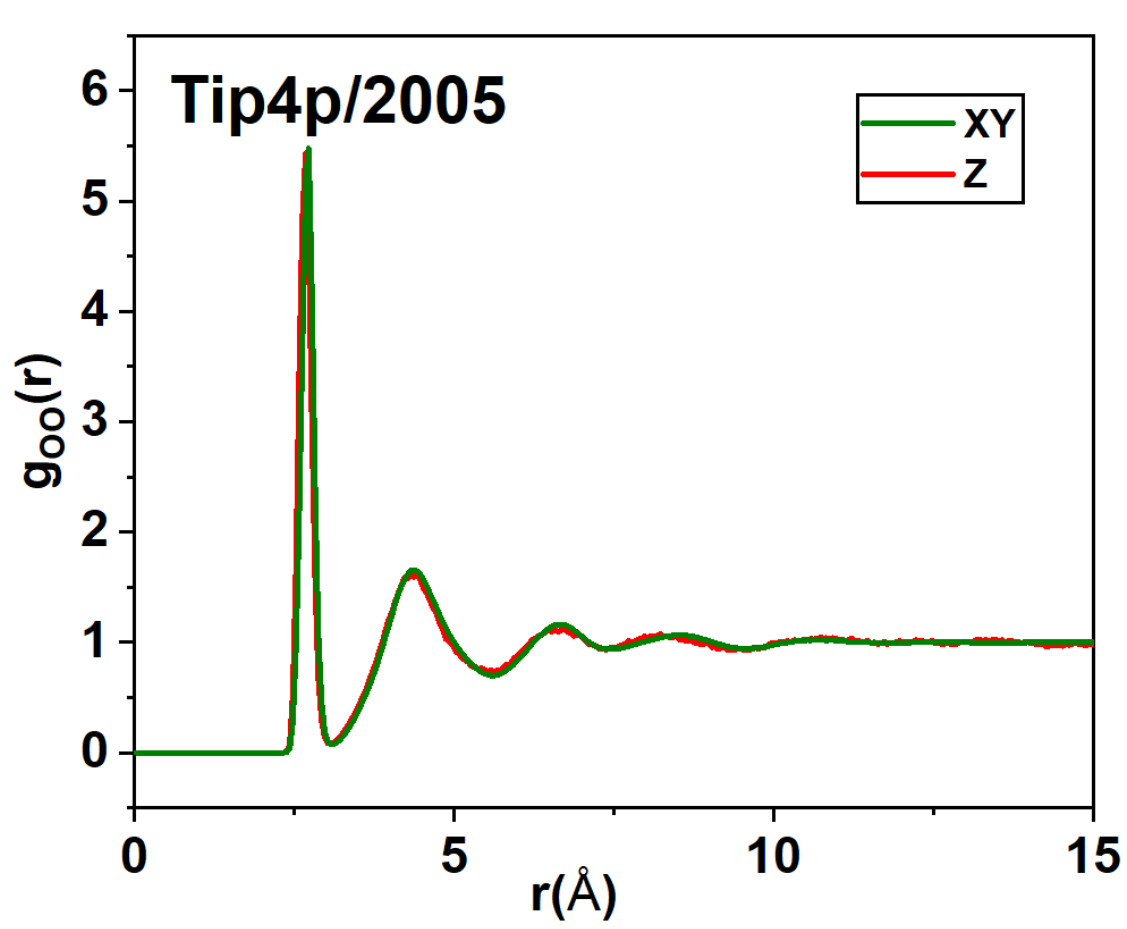

Figure S11. Structural anisotropy in the radial distribution function $g(r)$ from **MD simulations** for the configuration shown in Fig. S10c.

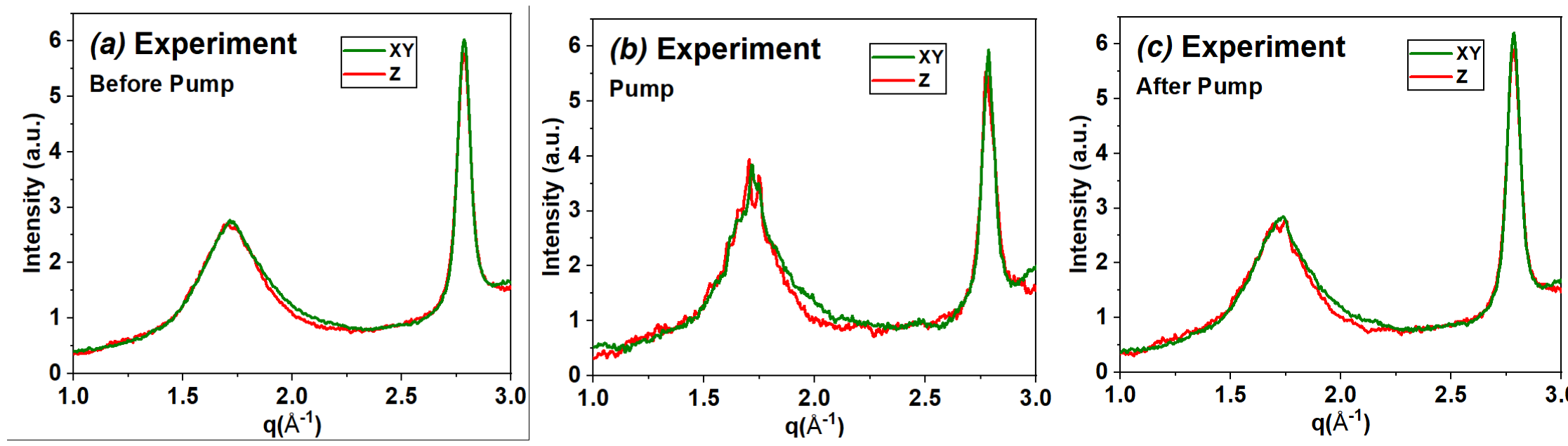

Figure S12. Representative **data from the multi-pulse mode**. Each sequence consists of one pump pulse (360 J/m²) followed by 30 X-ray probe pulses. Panel (a) shows the scattering signal before the pump pulse, panel (b) corresponds to the pumped state, and panel (c) shows the scattering after the pump pulse.

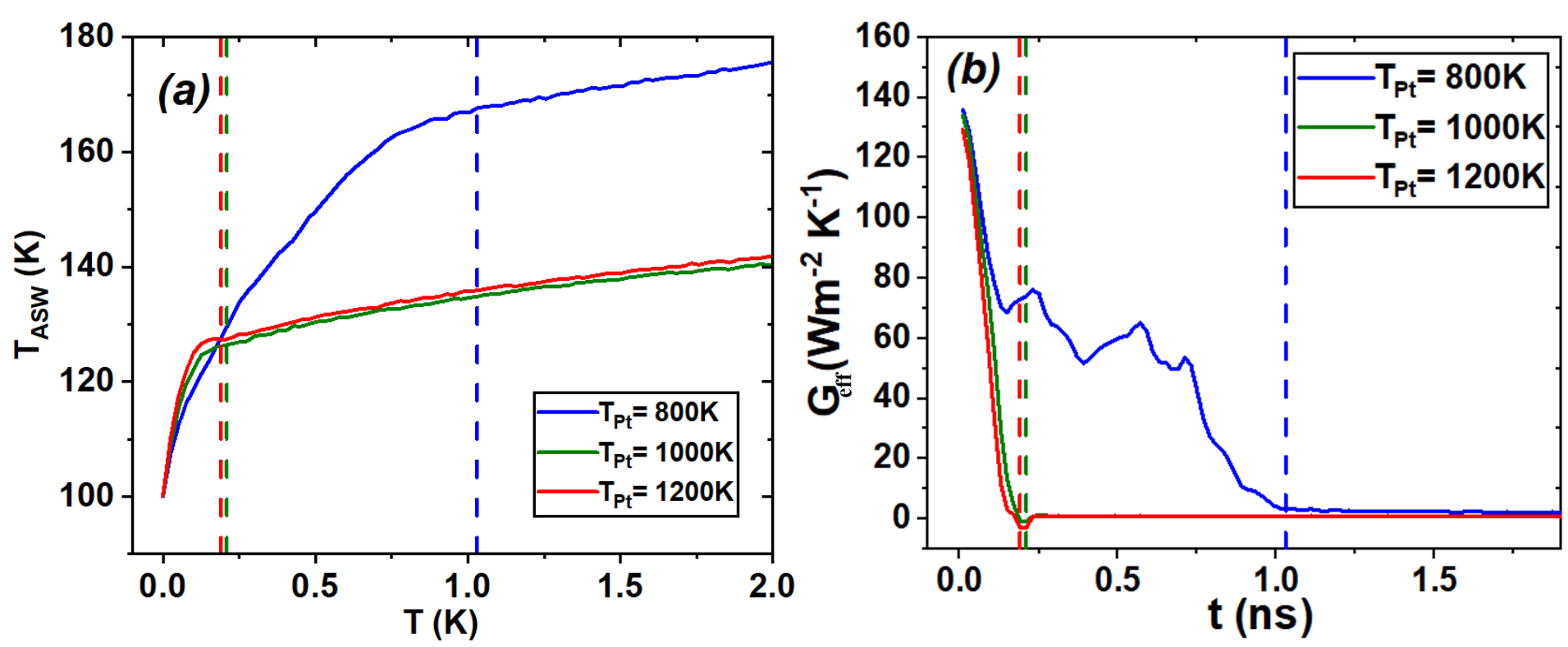

Figure S13. Transient temperature evolution and interfacial thermal conductance during Pt-induced heating of ASW, obtained using the **SPC/E water model**. (a) Temperature evolution of the 100-nm ASW film when the Pt substrate is held at fixed temperatures of 800 K, 1000 K, and 1200 K. The dashed lines indicate the approximate onset of nanoscale vapor-gap formation at the Pt–ASW interface. After the gap forms, the rate of temperature rise in the ASW decreases markedly, reflecting the strong suppression of heat transfer across the interface. (b) Interfacial thermal conductance $G_{eff}(t)$ extracted from the ASW energy flux and interfacial temperature difference. Before gap formation, $G_{eff}(t)$ remains at ∼130 MW m^{-2}K^{-1}, consistent with literature values for metal–water interfaces. Once the vapor gap develops, $G_{eff}(t)$ drops by 2–3 orders of magnitude to ∼0.4 MW m^{-2}K^{-1}, demonstrating the dramatic thermal decoupling.

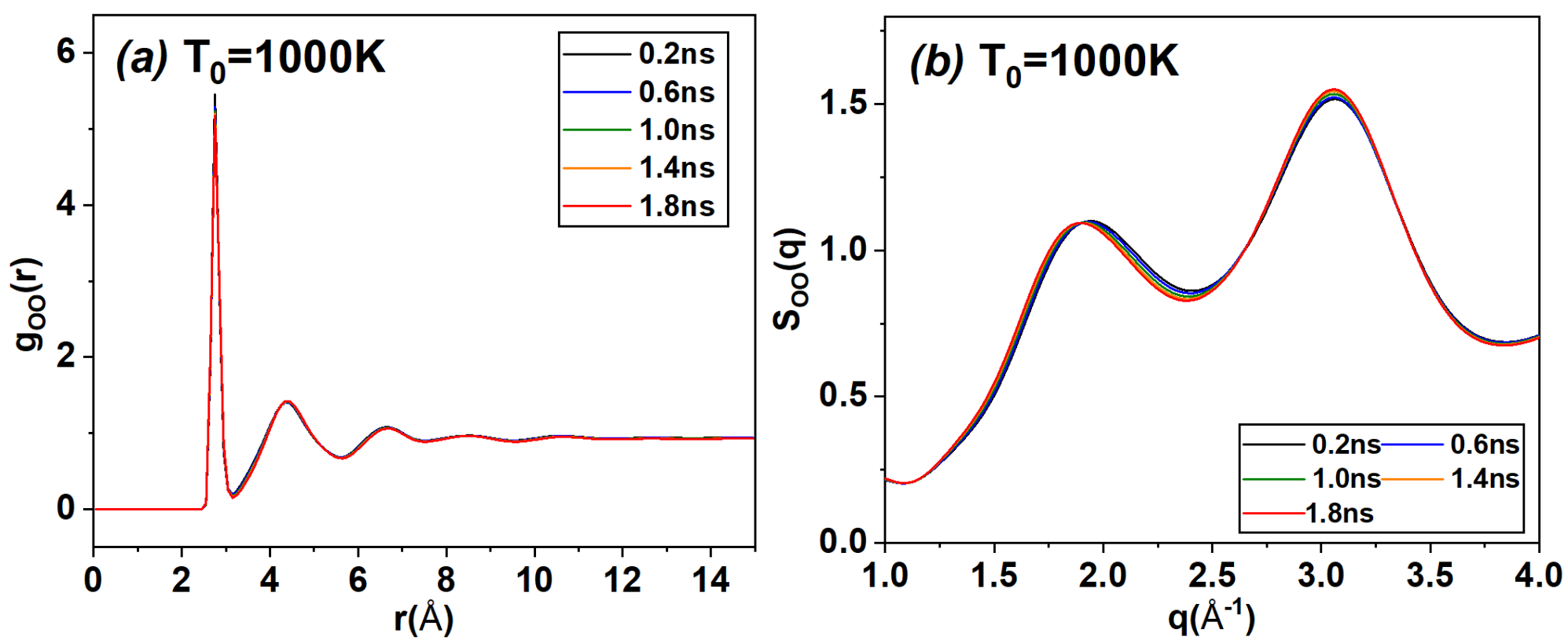

Figure S14. Time evolution of the structural response of ASW during ultrafast heating at an initial Pt temperature of T_0=1000 K. (a) Radial distribution function g(r) and (b) corresponding structure factor S(q) at selected time delays following substrate heating. A small but systematic shift of the first peak is observed at early times (sub-nanosecond timescale). In particular, the first peak in S(q) shifts from 1.94 $Å^{-1}$ to 1.90 $Å^{-1}$, corresponding to a relative decrease of approximately 2.1%, indicative of weak transient heating of the interfacial ASW prior to cavity formation. At later times, the structural evolution becomes suppressed, consistent with the formation of an interfacial vapor layer that limits further heat transfer into the ASW.

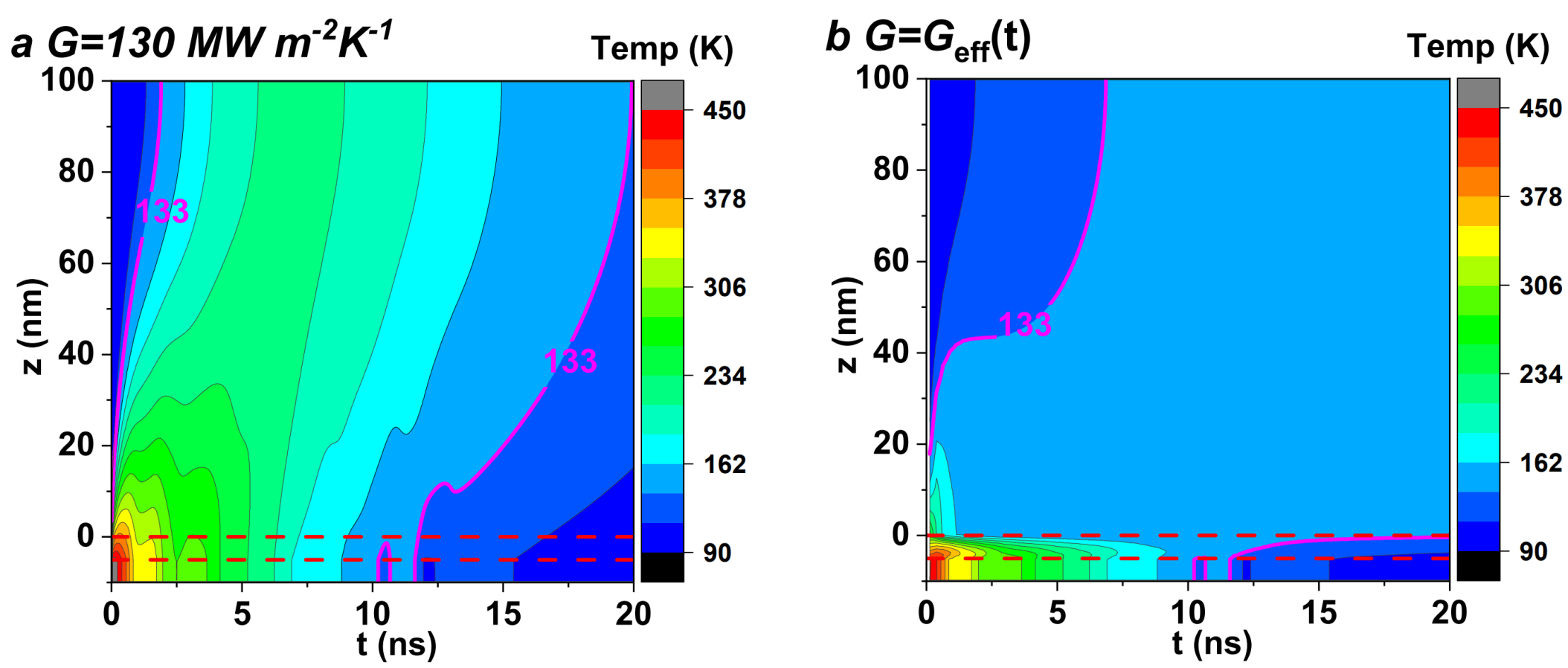

Figure S15. Effect of the interfacial thermal conductance on the **calculated temperature evolution** of the ASW film. Temperature profiles $T(z,t)$ obtained from the one-dimensional continuum heat-conduction model for the same Pt heating and cooling protocol. (a) Calculation using a constant interfacial thermal conductance of ($G \approx 130\ MW\ m^{-2}\ K^{-1}$). (b) Calculation using the time-dependent effective interfacial thermal conductance, ($G = G_{eff}(t)$), extracted from the MD simulation at $T_0 = 1000\ K$ shown in Fig. 6a. The rapid decrease in $G_{eff}(t)$ substantially suppresses heat transfer from Pt to the ASW film, whose temperature remains close to or below approximately 133 K. The thick magenta contour denotes the reference isotherm at $T = 133K$, close to the experimentally reported glass-transition temperature of ASW; it is included only as a reference for the propagation of the thermal front rather than as a precise non-equilibrium glass-transition boundary. The red dashed lines delimit the 5-nm interfacial region at the Pt–ASW boundary, within which the effective interfacial thermal conductance is implemented in the continuum model.

Figure S16. X-ray scattering from a bare Si–Pt substrate during cooling from 200 K to ~125 K taken prior vapor deposition. Each integrated curve is computed from 3000 XFEL pulses, corresponding to an acquisition window of 300 s at10 Hz, and normalized to the height of the first Pt reflection. The green curve at 125 K displays the accumulation of crystalline contamination below 125 K from residual background water at a vacuum level of 1.3 x 10^{-5} mbar.

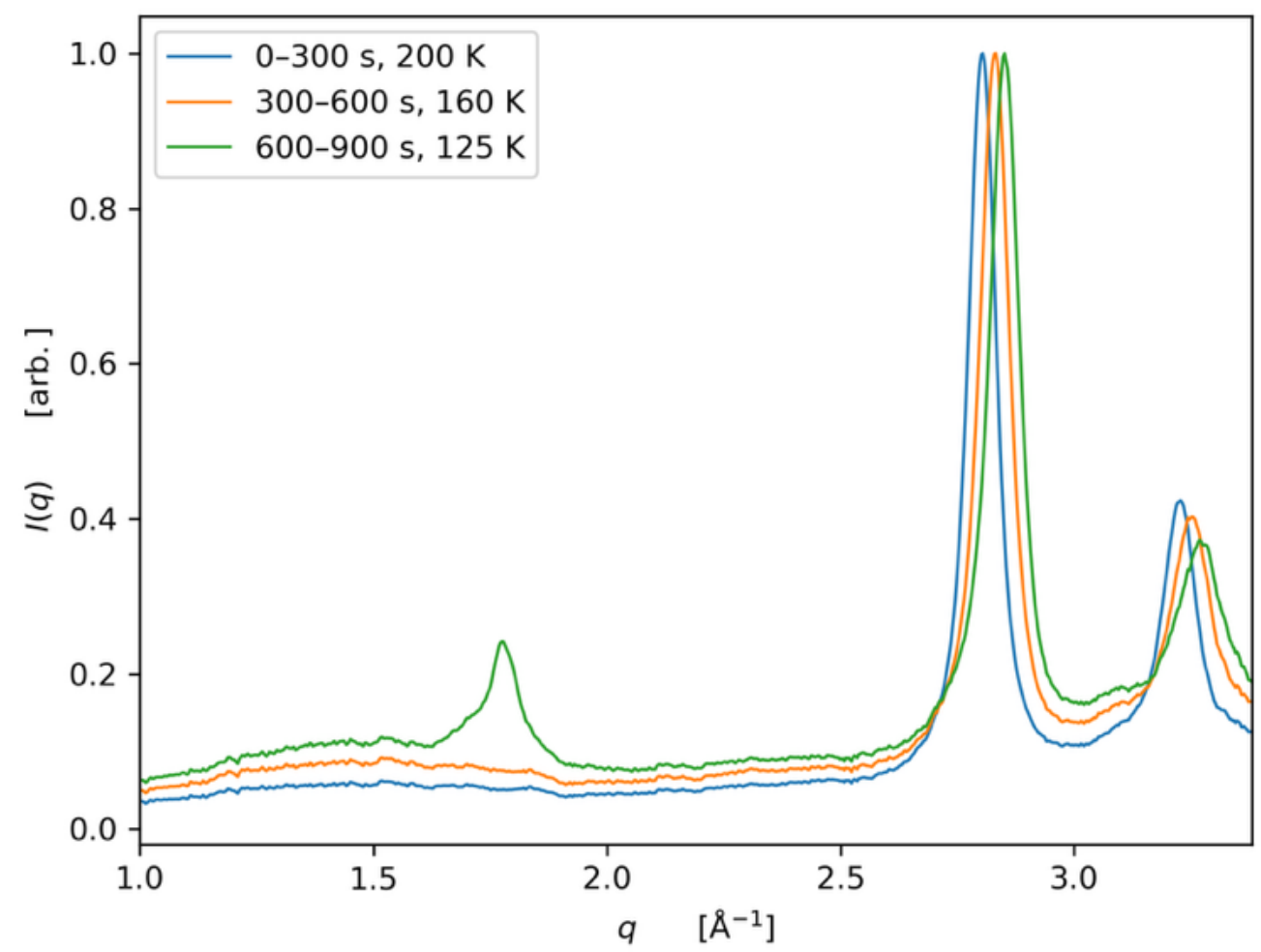

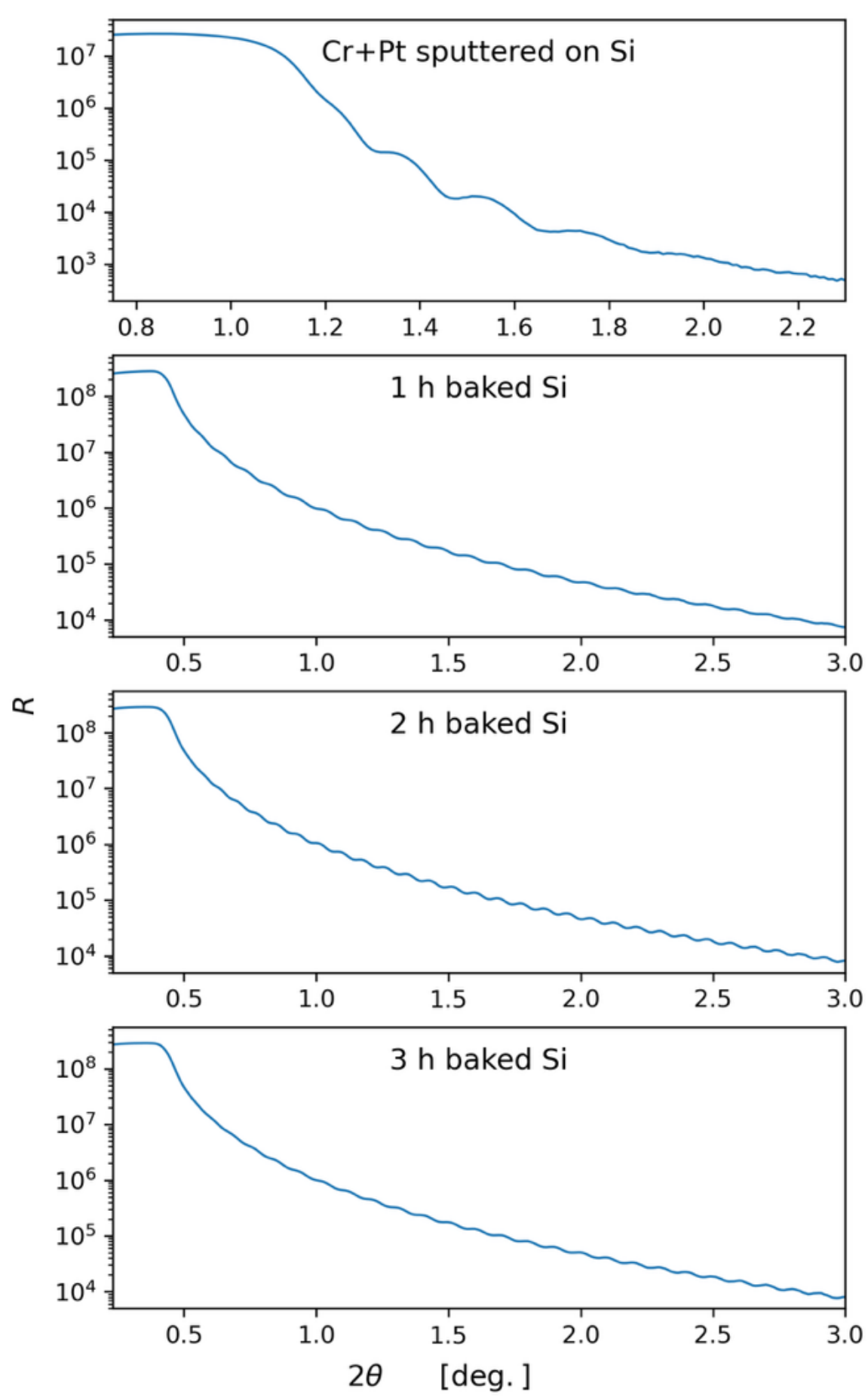

Figure S17. The ASW films were prepared by water vapor deposition of onto a platinum substrate, consisting of a 30 nm Pt layer on an oxidized Si wafer with an SiO_2 surface layer as well as a 5nm Cr buffer. Here we show the pre-characterization of the substrate (further details under Methods) using **X-ray reflectivity (XRR)**. The top panel shows reflectivity from a sputtered Pt surface, on top of a thin Cr buffer layer. The main oscillation corresponds to a layer thickness of approximately 30 nm. The three lower panels show reflectivity from baked Si wafers. The oscillations are caused by the SiO_2 oxide layer, which forms during baking at 1000°C. The oxide layer thicknesses are 76, 97, and 86 nm (1 h, 2 h, 3 h, respectively). Data was collected with a Rigaku SmartLab X-ray diffractometer using Cu K-α radiation (1.54 Å).

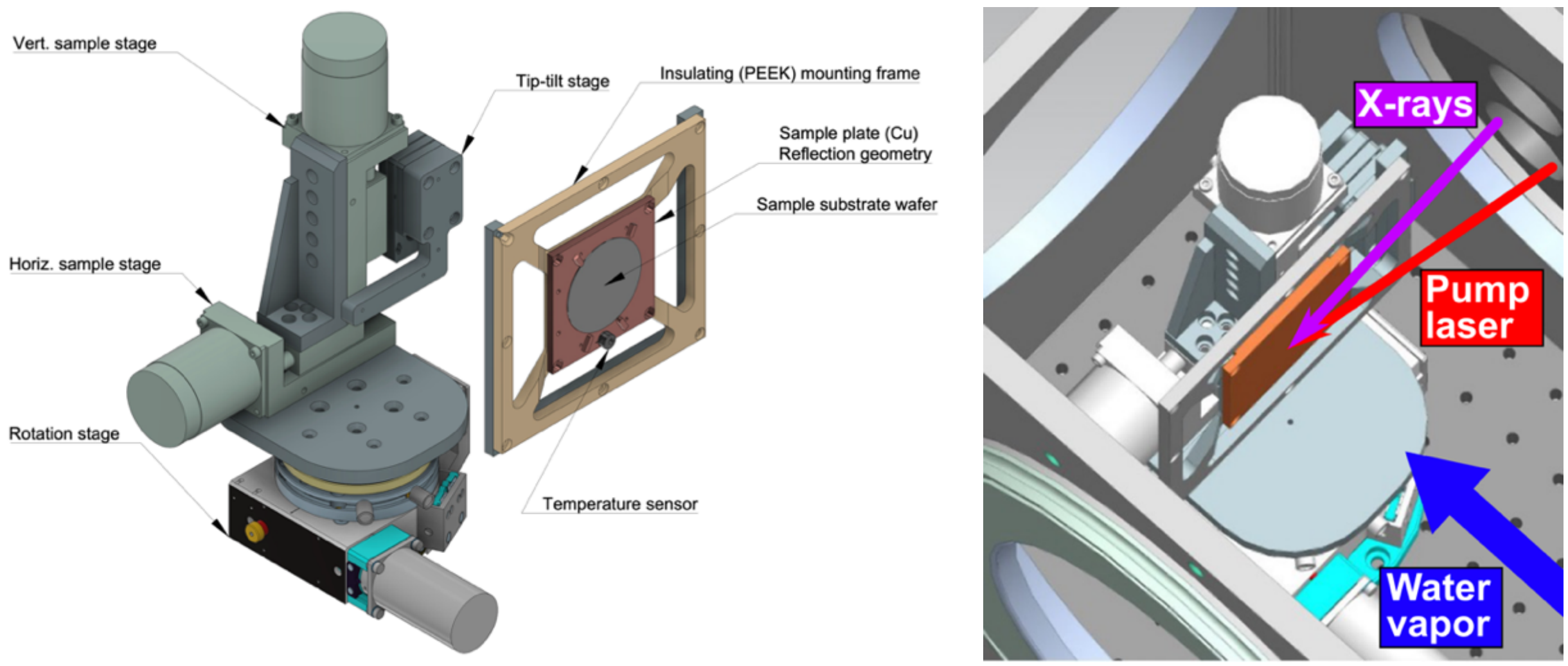

Figure S18. Experimental setup at FXE, European XFEL. Left: Sample goniometer and sample holder. The sample substrate is affixed with thermal paste and four leaf springs. The copper sample plate is thermally connected to a Janis SuperTran flow cryostat via a copper thermal strap (braid type). The 100 K sample temperature is measured with a PT100 resistance thermometer fixed to the sample plate.

Right: Scattering geometry inside the FXE vacuum chamber. The X-rays and pump laser are nearly co-linear with a crossing angle of ~1.1 °. The water vapor is injected through a 1/16 inch tube pointed directly at the substrate from a distance of 4 cm.

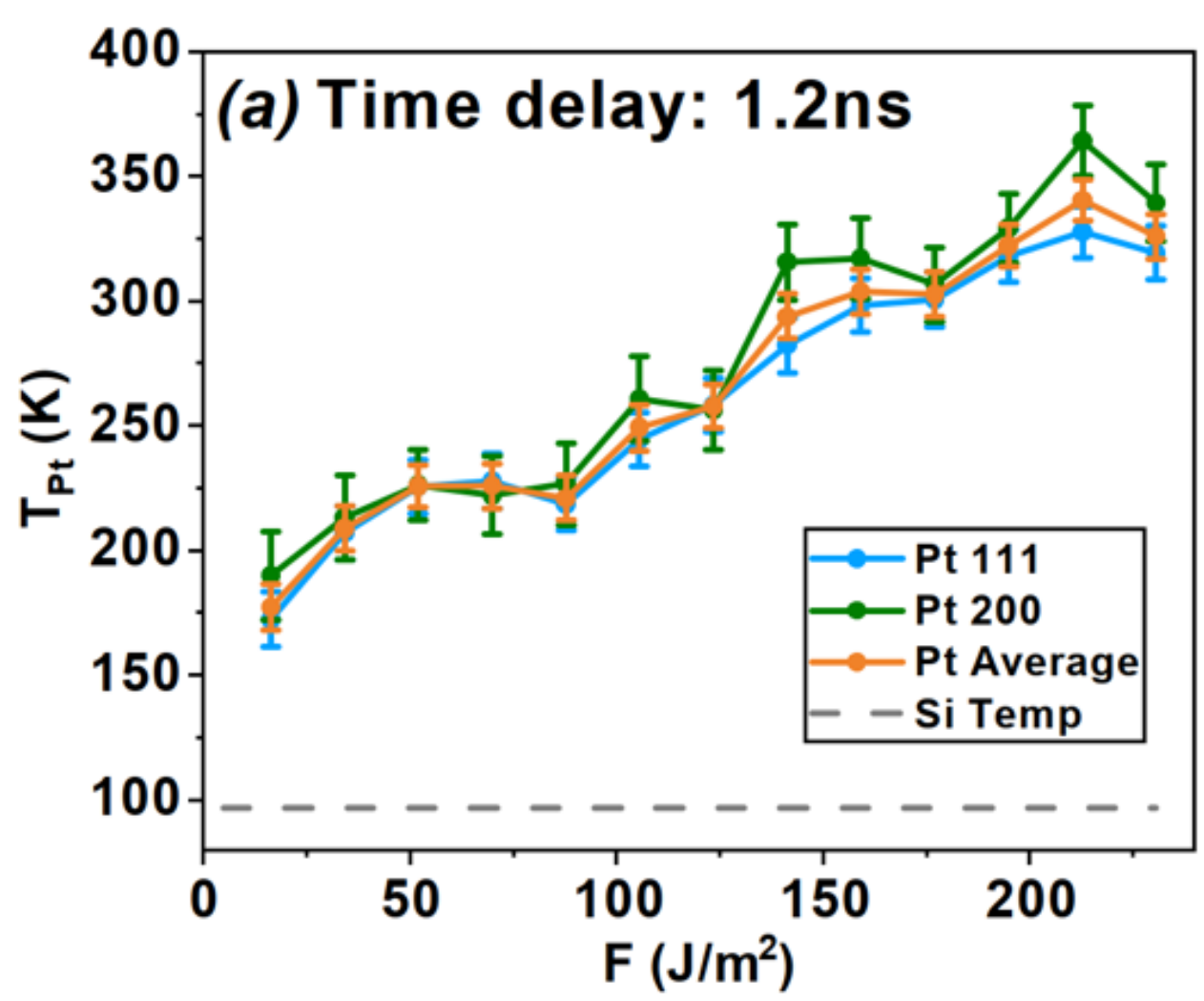

Figure S19. Experimentally determined evolution of the Pt temperature in fluences scan. $T(t)$ under different pump fluences at fixed pump-probe delay (1.2ns), showing the transient heating dynamics.

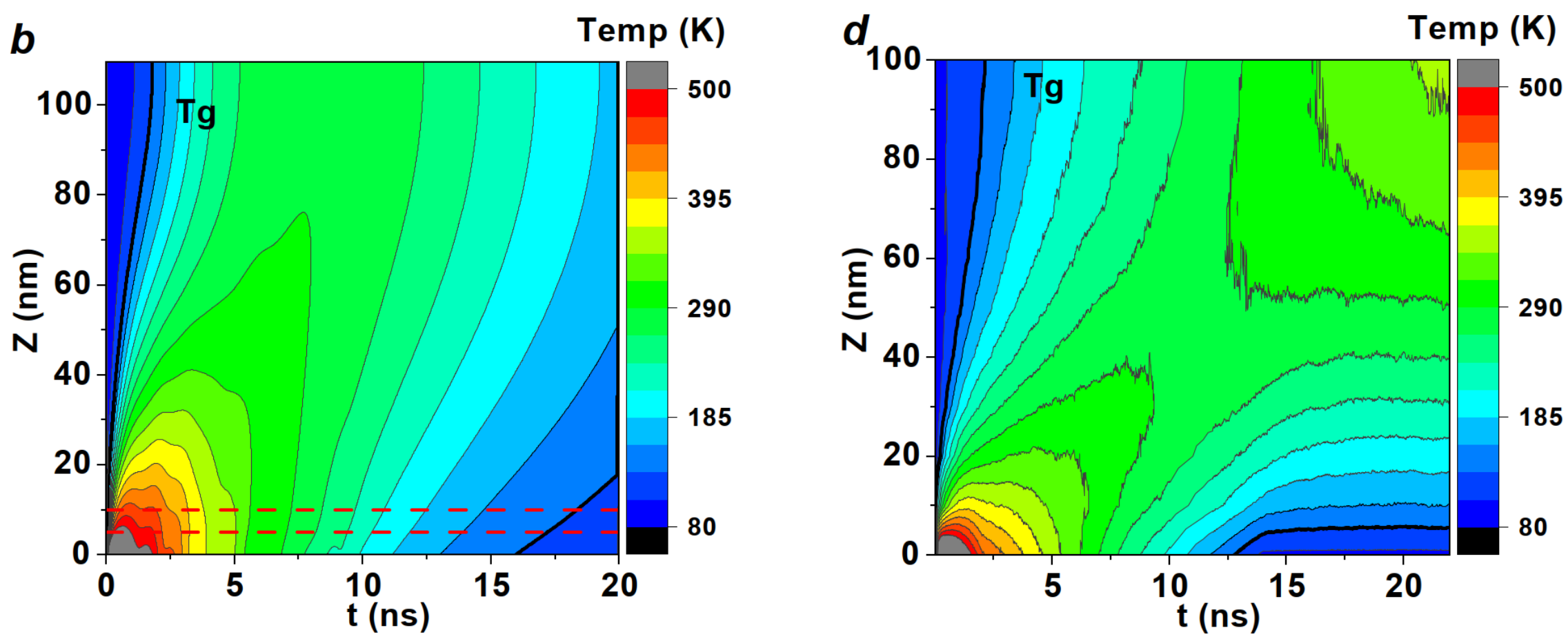

Figure S20. Temperature profiles $T(x,t)$ of ASW under different Pt heating protocols. **Continuum heat-conduction simulations** based on the one-dimensional thermal diffusion equation: (b) Pt heated to 750 K and then cooled. The temperature front propagates from the Pt–ASW interface into the film; the black contour marks the glass-transition isotherm ($T_g \approx 133$ K). (d) **Molecular dynamics simulations** (SPC/E model) under corresponding thermal conditions, showing similar evolution of temperature across the ASW film.

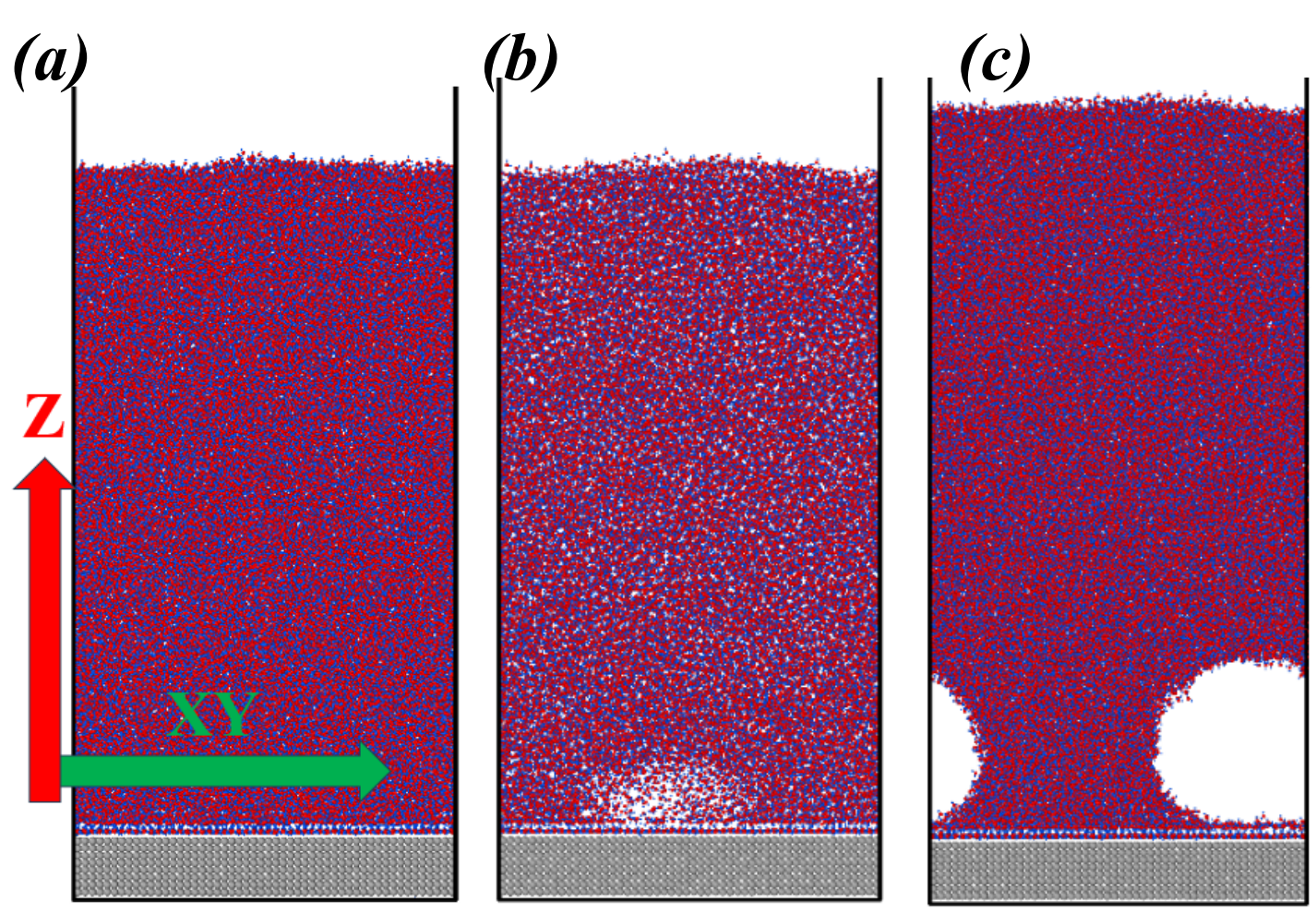

Figure S21. Representative atomic configurations with different vapor-gap thicknesses used for structure-factor calculations, obtained using the **SPC/E water model**. (a) Configuration without a vapor gap at the Pt–ASW interface, obtained by keeping the Pt slab fixed at 250 K, which suppresses the formation of the interfacial low-density layer. (b) Configuration with a ~2 nm vapor layer near the interface; only half of the simulation box along the x direction is shown for clarity, as the low-density region is located near the center of the system. (c) Configuration with a ~4.6 nm vapor layer at the interface.
